# Novel cleavage sites identified in SARS-CoV-2 spike protein reveal mechanism for cathepsin L-facilitated viral infection and treatment strategies

**DOI:** 10.1038/s41421-022-00419-w

**Published:** 2022-06-06

**Authors:** Miao-Miao Zhao, Yun Zhu, Li Zhang, Gongxun Zhong, Linhua Tai, Shuo Liu, Guoliang Yin, Jing Lu, Qiong He, Ming-Jia Li, Ru-Xuan Zhao, Hao Wang, Weijin Huang, Changfa Fan, Lei Shuai, Zhiyuan Wen, Chong Wang, Xijun He, Qiuluan Chen, Banghui Liu, Xiaoli Xiong, Zhigao Bu, Youchun Wang, Fei Sun, Jin-Kui Yang

**Affiliations:** 1 Department of Endocrinology, Beijing Diabetes Institute, Beijing Tongren Hospital, Capital Medical University, Beijing, China; 2 National Key Laboratory of Biomacromolecules, CAS Center for Excellence in Biomacromolecules, Institute of Biophysics, Chinese Academy of Sciences, Beijing, China; 3 Division of HIV/AIDS and Sex-Transmitted Virus Vaccines, Institute for Biological Product Control, National Institutes for Food and Drug Control (NIFDC), Beijing, China; 4 State Key Laboratory of Veterinary Biotechnology, Harbin Veterinary Research Institute, Chinese Academy of Agricultural Sciences, Harbin, China; 5 National High Containment Laboratory for Animal Diseases Control and Prevention, Harbin, China; 6 University of Chinese Academy of Sciences, Beijing, China; 7 Division of Animal Model Research, Institute for Laboratory Animal Resources, National Institutes for Food and Drug Control, Beijing, China; 8 Bioland Laboratory, Guangzhou, Guangdong China; 9 The State Key Laboratory of Respiratory Disease (SKLRD), Guangzhou Institutes of Biomedicine and Health, Chinese Academy of Sciences, Guangzhou, Guangdong China; 10 Center for Biological Imaging, Institute of Biophysics, Chinese Academy of Sciences, Beijing, China

**Keywords:** Cryoelectron microscopy, Mechanisms of disease

## Abstract

The spike (S) protein of severe acute respiratory syndrome coronavirus 2 (SARS-CoV-2) is an important target for vaccine and drug development. However, the rapid emergence of variant strains with mutated S proteins has rendered many treatments ineffective. Cleavage of the S protein by host proteases is essential for viral infection. Here, we discovered that the S protein contains two previously unidentified Cathepsin L (CTSL) cleavage sites (CS-1 and CS-2). Both sites are highly conserved among all known SARS-CoV-2 variants. Our structural studies revealed that CTSL cleavage promoted S to adopt receptor-binding domain (RBD) “up” activated conformations, facilitating receptor-binding and membrane fusion. We confirmed that CTSL cleavage is essential during infection of all emerged SARS-CoV-2 variants (including the recently emerged Omicron variant) by pseudovirus (PsV) infection experiment. Furthermore, we found CTSL-specific inhibitors not only blocked infection of PsV/live virus in cells but also reduced live virus infection of ex vivo lung tissues of both human donors and human *ACE2-*transgenic mice. Finally, we showed that two CTSL-specific inhibitors exhibited excellent In vivo effects to prevent live virus infection in human *ACE2-*transgenic mice. Our work demonstrated that inhibition of CTSL cleavage of SARS-CoV-2 S protein is a promising approach for the development of future mutation-resistant therapy.

## Introduction

The coronavirus disease 2019 (COVID-19) pandemic, caused by the novel SARS coronavirus 2 (SARS-CoV-2), has resulted in a global public health crisis. Most treatment strategies for SARS-CoV-2 infection are focused on vaccines or antiviral drugs targeting the viral spike (S) protein^1,2^ and the viral proteases, including the 3C-like protease (3CLpro, the main target of Paxlovid, Pfizer’s experimental COVID-19 pill^3^) and the papain-like protease^4^ (PLpro). However, the evolution of RNA viruses is driven by their high mutation rates, allowing their evasion of host immune attacks^5,6^. Despite multiple ongoing efforts to treat SARS-CoV-2 infections, variant strains with mutant spikes continue to emerge in many countries, causing new waves of infections with high morbidity^5–8^. Of note, the very recently emerged Omicron variant (B.1.1.529) has an unusually high number of mutations, with more than 30 in S protein, 1 in 3CLpro, and 5 in PLpro. Therefore, antiviral drugs or vaccines targeting the viral proteins are likely to encounter the problem of resistant mutations. Indeed, three vaccines (Janssen, Novavax, and AstraZeneca) tested against the Beta variant (B.1.351) exhibited reduced effects on preventing symptomatic infection, with effectiveness estimates of 57%, 49%, and even a statistically insignificant percentage, respectively^9^. The serum neutralizing activity against the Beta (B.1.351) variant among vaccinated persons was reduced by 6.5- and 8.6-fold for the BNT162b2 (Pfizer) and mRNA-1273 (Moderna) mRNA vaccines, respectively^10^. Molnupiravir is the chemical antiviral for the treatment of COVID-19 approved by the UK’s Medicines and Healthcare products Regulatory Agency (MHRA). However, the final study results Merck announced very recently raised questions about the drug’s benefit^11^. Thus, there is an urgent need to develop broad-spectrum and mutation-resistant treatment strategies for SARS-CoV-2 infection.

During coronavirus infection, multiple host cell proteases, such as furin, transmembrane serine protease 2 (TMPRSS2), and cathepsin L (CTSL)^12–14^ are known to process S protein; however, the exact sequence of protease cleavage and the interplay between host proteases remain poorly understood^15^. CTSL is a member of the lysosomal cysteine protease family, whose major function is proteolysis of antigens produced by pathogens. It is mainly detected in Golgi apparatus and traffic-related organelles like endosomes and lysosome^16^. It is highly expressed in most human tissues, including the respiratory system, gastrointestinal tract, kidney and urinary bladder, sexual tissues, bone marrow and lymphoid tissues, and endocrine tissues^17^. It was reported that SARS-CoV-2 can directly infect and injure many extrapulmonary organs, like the heart, kidney, liver, pancreas, and gastrointestinal tract^18–20^, usually with high CTSL expression levels. Besides, the mRNA expression level of CTSL is higher than angiotensin-converting enzyme 2 (ACE2), FURIN, and TMPRSS2 in human lung tissues^21^. Recently, we found that in SARS-CoV-2-infected patients, the circulating blood level of CTSL is highly correlated with the severity and course of COVID-19 symptoms. We also found that CTSL promotes SARS-CoV-2 pseudovirus (PsV) infection by cleaving the S protein and enhancing viral entry into cells^12^. However, the precise cleavage site and the mechanism by which CTSL activates the S protein remain uncharacterized. In addition, the effectiveness of CTSL inhibitors in preventing or treating infections of SARS-CoV-2 virus, remains unknown. In this study, we identified two specific cleavage sites for CTSL in SARS-CoV-2 S protein, which are highly conserved among all known SARS-CoV-2 variants. Mechanistic and functional studies revealed the mechanism by which CTSL cleavage is essential for efficient SARS-CoV-2 infection and inhibition of this process efficiently reduced virus infection. These results identify a new but promising target for broad-spectrum antiviral therapy development.

## Results

### CTSL cleaves the SARS-CoV-2 S protein at two specific sites

**Fig. 1:**
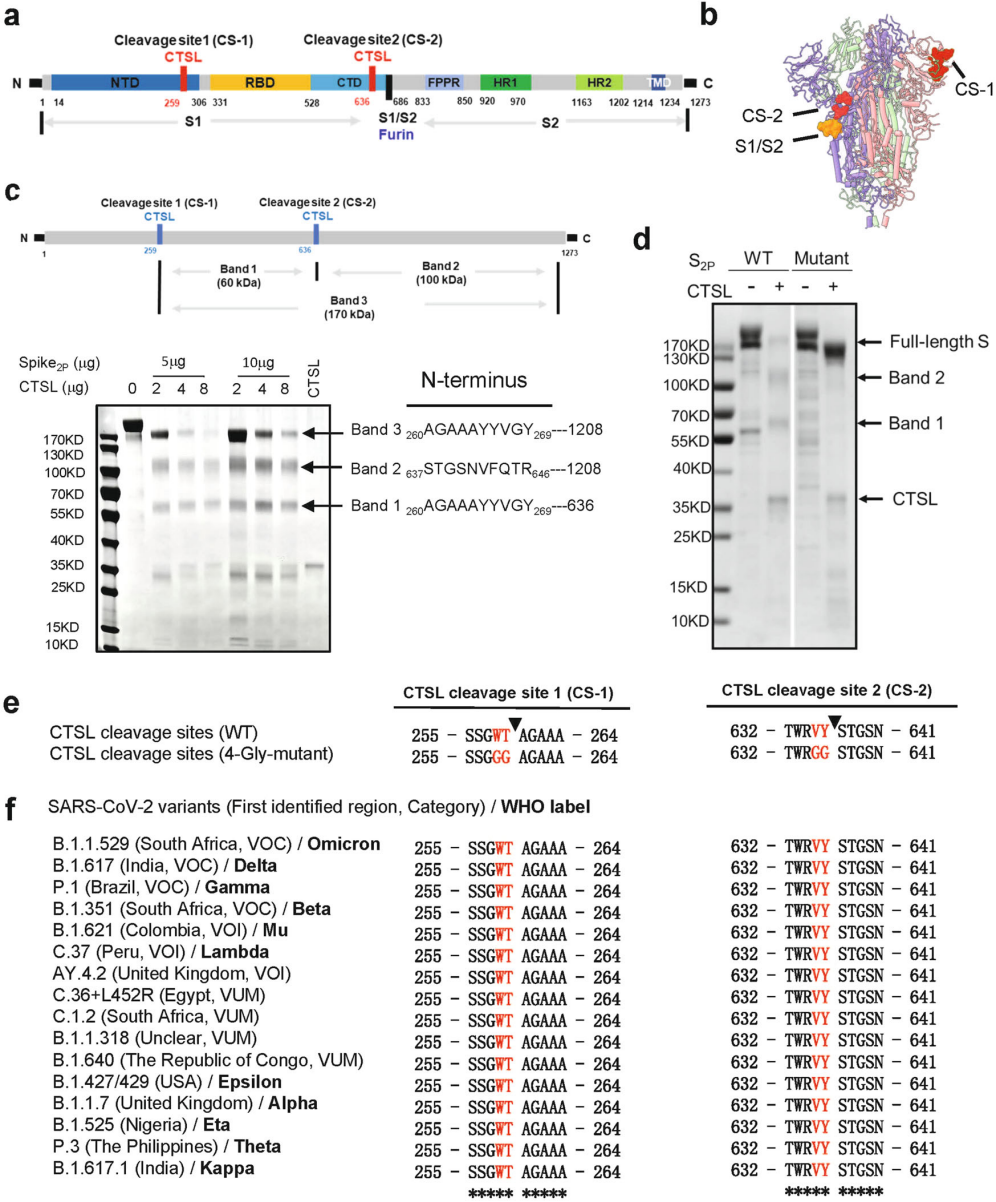
CTSL cleaves the SARS-CoV-2 S protein at two novel sites. **a** Schematic illustration of the SARS-CoV-2 S glycoprotein in which the functional domains and cleavage sites are highlighted (NTD N-terminal domain, RBD receptor-binding domain, CTD C-terminal domain, FPPR fusion-peptide proximal region, HR1 heptad repeat 1, HR2 heptad repeat 2, TMD transmembrane domain). CTSL cleaves at CTSL cleavage site 1 (CS-1) and CTSL cleavage site 2 (CS-2). Furin cleaves at the S1/S2 site. **b** Overall structure of the SARS-CoV-2 S ectodomain (PDB entry: 6VXX). The CTSL cleavage sites, CS-1 and CS-2, are colored in red, while the furin cleavage site at S1/S2 is colored orange. The three protomers of the S trimer are colored in pink, purple and green. **c** Schematic illustration and SDS-PAGE analysis for the cleavage of SARS-CoV-2 S glycoprotein. The purified SARS-CoV-2 S protein ectodomain was incubated with different concentrations of CTSL (2–8 μg/mL). The N-terminal sequencing results for band 1 (60 kDa), band 2 (100 kDa), and band 3 (170 kDa) are also shown. All samples were subjected to SDS-PAGE, and bands were detected by Coomassie blue staining. **d** 4-Gly-mutant SARS-CoV-2 S cannot be cleaved by CTSL into the 60 kDa (band 1) and 100 kDa (band 2) fragments. WT S_2p_ protein (1 μg) and mutant S protein (1 μg) were incubated with CTSL (8 μg/mL). All samples were subjected to SDS-PAGE, and bands were detected by silver staining. **e** The P1 and P2 residues in CS-1 and CS-2 were mutated to glycine, and the mutant SARS-CoV-2-2-S glycoprotein was named 4-Gly-mutant S. **f** Amino acid sequence alignment of residues around CS-1 and CS-2 in SARS-CoV-2 variants. P1 and P2 residues that are the same as the WT residues are highlighted in red. The symbol * indicates amino acid residues that are conserved among all tested sequences. VOC variant of concern, VOI variant of interest, VUM variant under monitoring.

The trimeric S protein that incorporated into the SARS-CoV-2 envelope contains S1 and S2 subunits (Fig. 1a, b). The S1 subunit binds to the host cellular receptor, while the S2 subunit is involved in the virus-cell membrane fusion process^22^, which is followed by the release of viral genetic material into target cells^23^. To study the structural and functional changes in the S protein upon treatment with CTSL, we cloned the mammalian codon-optimized nucleotide sequence encoding the SARS-CoV-2 (Wuhan-Hu-1 strain, GenBank ID: MN908947.3) S protein ectodomain (residues M1–Q1208) with proline substitutions at K986 and V987 and a “GSAS” substitution at the furin cleavage site (S1/S2 site, R682–R685) and purified the resulting protein, named S_2P_ protein. This mutant S protein is widely used for structural and functional analysis due to its enhanced stability^24^. After co-incubation with CTSL, the purified S protein was cleaved into three major fragments, and the reaction exhibited a dose-dependent relationship with the CTSL concentration (Fig. 1c). Based on N-terminal amino acid sequencing of these three fragments, we identified two novel CTSL cleavage sites of S, 259 T (named CTSL cleavage site 1 or CS-1) and 636Y (named CTSL cleavage site 2 or CS-2) (Fig. 1c; Supplementary Fig. S1a–c), which were further confirmed by liquid chromatography mass spectrometry (LC-MS) analysis (Supplementary Fig. S1d, e). CS-1 is located in the N-terminal domain (NTD) of the S1 subunit, while CS-2 is located in the C-terminal domain (CTD) of the S1 subunit (Fig. 1a). Similar to the furin cleavage site at S1/S2, both CTSL cleavage sites are located in the exposed loops of the prefusion S protein, which are accessible to proteases (Fig. 1b). We noted that a recent publication^25^ analyzed the endogenous proteolysis sites of S protein during SARS-CoV-2 infection by mass spectrometry and identified many cleavage sites, including 260 A and 637 S. However, they did not make further assignments of the identified cleavage sites to specific proteases. Our work by discovering the CTSL cleavage sites correlates precisely with their work and provides the exact explanation of their data. As a result, we are more confident that the CTSL cleavage plays a very important role during the real SARS-CoV-2 infection process.

To verify these two cleavage sites, we generated a mutant with mutations at the sites of CS-1 and CS-2. It has been reported that substrate recognition by CTSL is determined mainly by the cleavage site residue (named the P1 residue) and its adjacent upstream residue (named the P2 residue)^26^. Therefore, we mutated both the P1 and P2 residues in CS-1 and CS-2 to glycine to generate a 4-Gly-mutant of S (Fig. 1e). As expected, treatment of the mutant with CTSL did not yield the two cleaved fragments, confirming the cleavage specificity at the sites of CS-1 and CS-2 (Fig. 1d).

### CS-1 and CS-2 sites are highly conserved among SARS-CoV-2 variants

To date, many SARS-CoV-2 variants have been found in different countries (Fig. 1f). These variants exhibit enhanced transmission, pathogenicity, immune escape, or a combination of all three, and different countries can have different dominant variants^27^. Variant viruses always encoded spike proteins with substitutions in antibody binding hot spots. In contrast, our newly identified CTSL cleavage sites in S protein are highly conserved in SARS-CoV-2 variants (Fig. 1f), including the recently emerged Omicron variant, suggesting that these CTSL cleavage sites may be essential for the SARS-CoV-2 life cycle.

It has been reported that SARS-CoV-2 may originate from bat coronaviruses^28^, such as RaTG13. Further sequence analysis of SARS-CoV-related coronaviruses (SARSr-CoVs) found in humans, bats, and pangolins showed that the CS-2 site is highly conserved among most SARSr-CoVs while the site of CS-1 is conserved only among SARS-CoV-2 like CoVs (Supplementary Fig. S2a). It suggests that the two CTSL cleavage sites may evolve to fulfil different functional roles in different SARSr-CoVs. The highly conserved CS-2 site seems to be more essential for the life cycle of SARSr-CoVs, while CS-1 is likely to play an auxiliary role in virus infection. Moreover, we compared the conservation of the two CTSL cleavage sites among seven known human CoVs and found that these CTSL cleavage sites exist only in SARS-CoV-1 and SARS-CoV-2 (Supplementary Fig. S2b), suggesting that CTSL would play a unique role in the process of SARS-CoV-1/2 infection. This observation is consistent with previous studies showing that CTSL is also involved in SARS-CoV-1 infection^29^.

### CTSL cleavage activates SARS-CoV-2 S protein

To investigate how CTSL cleavage affects the structure of SARS-CoV-2 S, we performed cryo-EM studies of both the CTSL-treated and untreated S protein. The double-proline substitutions have been shown to affect S protein conformation and dynamics. Therefore, to further study the structural changes close to the natural state of S protein, we expressed and purified SARS-CoV-2 S protein ectodomain named S_R_ with minimum modifications^30^. Compared to the full-length S protein, S_R_ protein contains the ectodomain (residues M1–Q1208) of S with a foldon at the C-terminus, and the furin cleavage site (P681 to R685) at the S1/S2 junction is replaced with a single arginine (R) resembling that in SARS-CoV-1 S to avoid the cleavage by furin during protein expression. The expression tags of trimeric foldon were removed by Tobacco Etch Virus protease (TEV) cleavage before our experiments (Supplementary Fig. S3a). We also confirmed that the cleavage pattern of S_R_ by CSTL is almost the same as that of S_2P_ (Supplementary Fig. S3b).

**Fig. 2:**
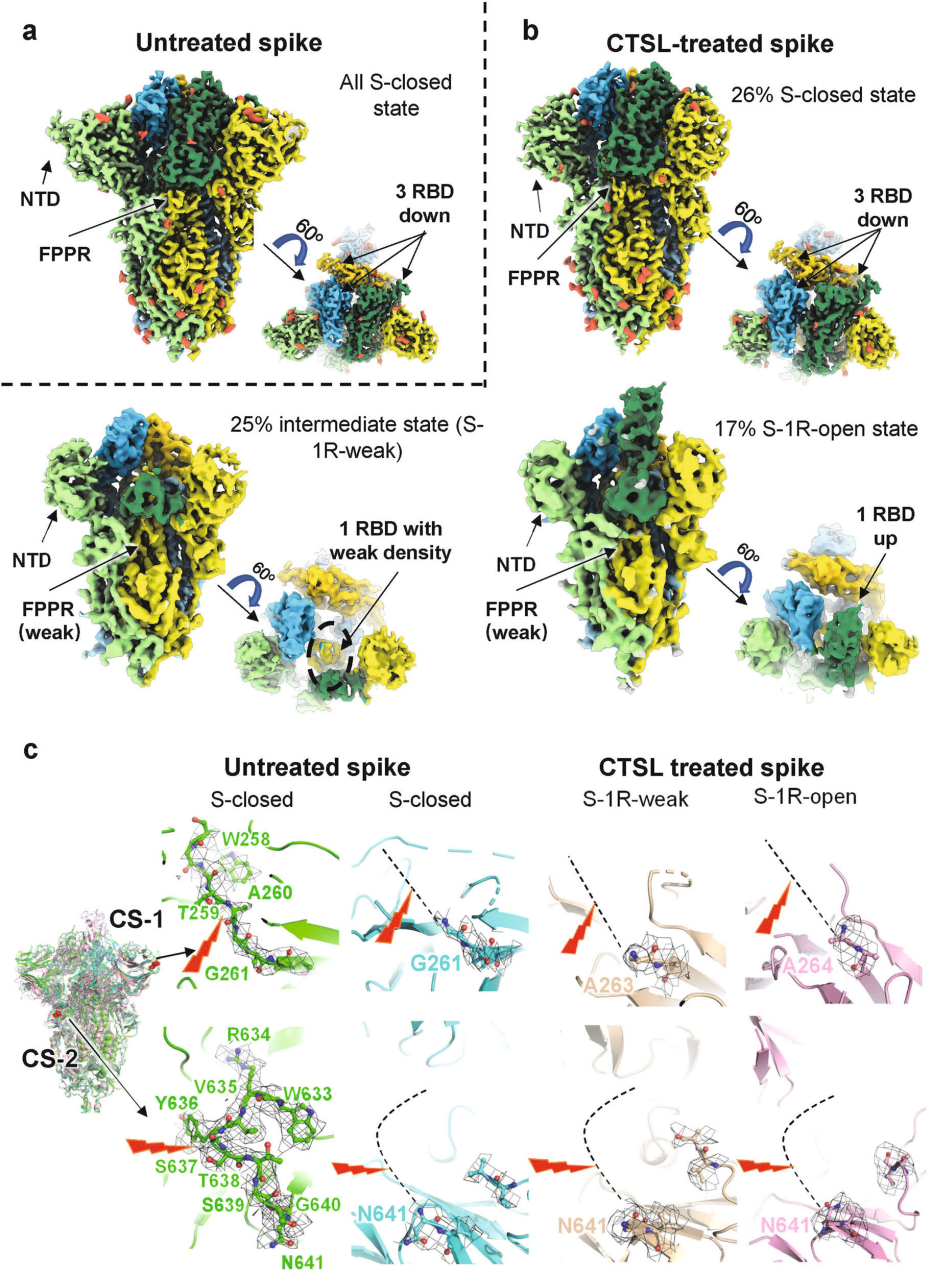
Cryo-EM structures of CTSL-untreated and -treated SARS-CoV-2 S proteins. **a** Side and top views of cryo-EM map of untreated S protein. The three protomers of S protein are shown in light green, yellow, and sky blue. The three RBD domains are highlighted in Sea Green, gold, and deep sky blue, respectively. Glycosylation modifications are colored in tomato. **b** Side and top views of cryo-EM map of CTSL-treated S protein in different states. The map colors are as same as **a**. **c** The four structures of S proteins are superposed together in different colors. CS-1 and CS-2 sites are colored in red and as indicated. The CS-1 and CS-2 regions are zoomed in to show the details. The cleavage sites are indicated by the red lighting-shaped symbol. For untreated S protein, the electron density around the cleavage site is represented as black grid. For the CTSL-treated S, the cleavage site cannot be traced and is represented as dotted line.

To ensure the comparability in the subsequent cryo-electron microscopy (cryo-EM) structural analysis, the samples in CTSL-treated group and control group were operated under the same condition, and the collected cryo-EM data were processed in the same workflow (Supplementary Fig. S4). In the untreated control group, all the particles fell into one population of the closed state (named S-closed) (Fig. 2a; Supplementary Figs. S4a and S8a), with a resolution of 3.2 Å in C3 symmetry according to the gold standard Fourier shell correlation coefficient at 0.143 (Supplementary Figs. S5 and S6). In this closed state, the surface of the receptor-binding domain (RBD) is buried inside the trimer and is not accessible for the receptor ACE2 binding^22,31^. The NTD and fusion-peptide proximal region (FPPR) are highly ordered in this state, resembling the “locked” closed conformation that was previously reported in multiple studies using full-length or ectodomain S proteins^30–34^.

However, after CTSL treatment, the S particles fell into four different populations, including one closed state (S-closed, 26%) and three activated states, named S-1R-weak (25%), S-1R-open (17%), and S-2R-1N-weak (32%) (Supplementary Fig. S4b). Beside the S-2R1N-weak state (Supplementary Fig. S8b), the full pseudoatomic structural models for the other three states were built at a resolution range of 3.5–4.5 Å (Supplementary Figs. S5 and S6).

Except for the truncated NTD, the structure of S-closed state in CTSL-treated group is almost identical to that in the control group with a root mean square deviation (RMSD) value of only 0.677 Å (Fig. 2b; Supplementary Table S1). For the S-1R-weak state (also known as intermediate state), one of the three RBDs has a very weak density, suggesting that this RBD exposes from the buried surface and becomes dynamic for ACE2 binding (Fig. 2b)^35^. The RMSD value between the rest part of S-1R-weak state and the S-closed state in CTSL-treated/control group is 0.899/1.277 Å, respectively (Supplementary Table S1). For the S-1R-open state, one of the three RBDs becomes upright and fully exposed for ACE2 binding (Fig. 2b)^31^, with RMSD values of 1.083/1.122 Å in comparison with the S-closed state in CTSL-treated/control group, respectively (Supplementary Table S1). We also noted that both NTDs in S-1R-weak and S-1R-open states are also truncated in comparison with the S-closed state of the control group, similar to the phenomenon observed for the S-closed state after CTSL treatment. The FPPR regions in S-1R-weak and S-1R-open states both have very weak densities, also suggesting that the S proteins at these two states are activated^32^. Moreover, the biolayer interferometry (BLI) experiment was performed to examine the interaction between S protein and human ACE2 receptors before and after CTSL cleavage (Supplementary Fig. S7). The results showed that CTSL treatment increased the binding affinity between S protein and ACE2 receptor, suggesting the activation of S protein by CTSL.

For the S-2R1N-weak state, although the resolution is too low to build the pseudoatomic model (Supplementary Fig. S8b), its functional domains, including NTD, C-terminal domain (CTD), and RBD, could be clearly distinguished from the cryo-EM map. In this state, two RBDs and one NTD all have very weak densities. We postulated that this state might represent a state in which S1 is dissociating to expose S2 subunit for membrane fusion.

Compared with the control group, the proportion of the inactive state population (S-closed) was significantly reduced from 100 to 26% after the cleavage by CTSL (Fig. 2b), suggesting that CTSL cleavage induces significantly increased dynamics of the RBDs and NTDs with an increased proportion of activated S.

### Structural evidence for CTSL cleavage site

Although the overall structures of S trimer before and after CTSL treatment are basically the same (Fig. 2c), they have significant structural differences in the local regions around CS-1 and CS-2 sites. For the CS-1 region, residues from S255 to G261 can be clearly traced in the density map and the cleavage site of CS-1 between T259 and A260 can be modeled with high confidence before CTSL treatment (Fig. 2c). However, after CTSL treatment, all the densities around CS-1 site disappeared, and we can only build as far as G261 in the models of S-closed state, and A263/A264 in the S-1R-weak/S-1R-open state (Fig. 2c). These results confirmed the occurrence of CTSL cleavage around the CS-1 site.

For the CS-2 region, residues from W633 to N641 can be clearly traced in the density map and the cleavage site of CS-2 between Y636 and S637 can be modeled with high confidence before CTSL treatment (Fig. 2c). However, after CTSL treatment, all the densities around CS-2 site disappeared, and we can only build as far as N641 in the models of S-closed, S-1R-weak and S-1R-open states. These results also confirmed the occurrence of CTSL cleavage around the CS-2 site.

### CTSL cleavage is essential for SARS-CoV-2 infection and cell fusion

**Fig. 3:**
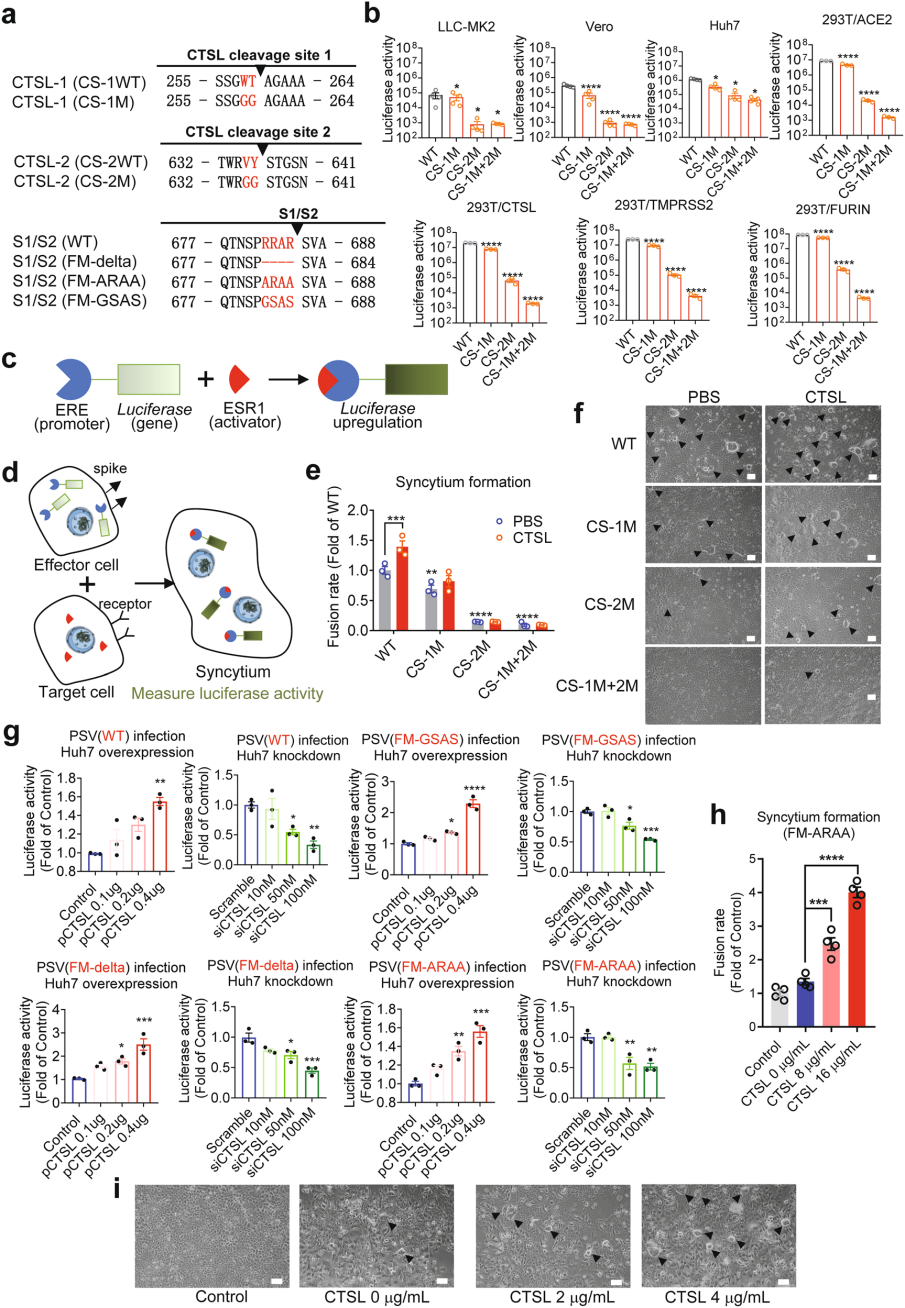
CTSL cleavage sites are essential for SARS-CoV-2 infection and efficient cell–cell fusion. **a** Overview of SARS-CoV-2 S proteins with mutations in CS-1, CS-2, and the S1/S2 cleavage site. **b** Infectivity of PsVs with different point mutations in CS-1 and CS-2 was assessed in LLC-MK2, Vero, Huh7, 293 T/ACE2 cells and in 293 T/ACE2 cells with CTSL (293 T/CTSL), TMPRSS2 (293 T/TMPRSS2), and FURIN (293 T/FURIN) genes overexpression. PsV infectivity was measured by a luciferase assay and is shown as the raw luciferase activity (*n* = 3–4). Statistical significance was assessed by one-way ANOVA with Tukey’s post-hoc test. **c–e** Quantitative analysis of syncytium formation induced by CS mutant SARS-CoV-2 S proteins (see also Supplementary Fig. S9). **c** Luciferase gene expression was driven by the ERE promoter, and ESR1 (activator) bound and activated the ERE promoter to upregulate luciferase expression. **d**, **e** Effector Huh7 cells were cotransfected with plasmids expressing ERE-luciferase and different S proteins as indicated, and another plate of target Huh7 cells was transfected with plasmid expressing ESR1. After 24 h, the effector cells were detached and added to the target cells for 30–60 min. Then, the supernatant was removed and treated with PBS or CTSL (8 μg/mL) for 20 min. The reaction was stopped by adding 500 μL of medium, and culture was continued for another 24 h to allow cell–cell fusion. When a target cell and effector cell fused to form a syncytium, ESR1 bound and activated the ERE promoter to upregulate luciferase expression. Luciferase activity was then measured as a proxy for the fusion rate. The data were normalized to the WT-PBS group (*n* = 3). Statistical significance was assessed between the indicated group and the WT-PBS group by two-way ANOVA with Dunnett’s post-hoc test. **f** Images of syncytium formation induced by CS mutant SARS-CoV-2 S proteins. Huh7 cells were transfected with plasmids to express the WT, CS-1M, CS-2M, or CS-1M + 2 M S protein. Cells were treated in the absence (PBS, pH = 5.8) or presence of CTSL (4 μg/mL, pH = 5.8). Images were acquired after an additional 10–16 h of incubation in medium (scale bars, 50 μm). The black arrowheads indicate syncytia. Representative data from three independent experiments are shown. **g** Overexpression or knockdown of the *CTSL* gene dose-dependently promoted or inhibited, respectively, infection with WT (Wuhan-1) and three mutant SARS-CoV-2 PsVs with different point mutations in the furin cleavage site (FM-delta, FM-ARAA, and FM-GSAS). PsV infectivity in Huh7 cells was measured by a luciferase assay and is shown as the relative luciferase activity (*n* = 3). Statistical significance was assessed by one-way ANOVA with Tukey’s post-hoc test. **h** CTSL promoted syncytium formation induced by the FM-ARAA mutant SARS-CoV-2 S protein. Effector cells were cotransfected with ERE-luciferase plasmids and either FM-ARAA S or scramble vectors (Control). Target cells were transfected with ESR1 expression plasmid. After the effector cells and target cells were mixed, the supernatant was removed and treated with PBS or CTSL (8 μg/mL and 16 μg/mL). Luciferase activity was then measured and normalized to that in the control group (*n* = 4). Statistical significance was assessed by one-way ANOVA with Tukey’s post-hoc test. **i** Huh7 cells were transfected with scramble vector (Control) or FM-ARAA S protein expression plasmid. Cells were treated in the absence or presence of CTSL (2 or 4 μg/mL). Images were acquired after an additional 10–16 h of incubation in medium (scale bars, 50 μm). The black arrowheads indicate syncytia. Representative data from four independent experiments are shown. The data are presented as the means ± SEM. *P < 0.05, **P < 0.01, ***P < 0.001, ***P < 0.0001.

Our structural studies above suggested the important role of CTSL cleavage in the activation of S protein. To verify these observations, we investigated the functions of CTSL during viral infection. We generated several mutants of PsVs based on the two cleavage sites (CS mutant), including CS-1 mutant (CS-1M), CS-2 mutant (CS-2M), and combination mutant (CS-1M + 2 M) (Fig. 3a). The binding affinity for hACE2 was not reduced in the CS mutant spike (CS-1M + 2 M) compared with the control S_2p_ (Supplementary Fig. S9a). As the proper control, we generated three PsV mutants that have been proven to escape furin cleavage: furin mutant (FM)-delta (deletion of residues 682–685), FM-ARAA (_682_ARAA_685_), and FM-GSAS (_682_GSAS_685_). The expression level of S protein in different PsVs was comparable (Supplementary Fig. S9b).

Then, we compared the infectivity of wild-type (WT) and mutant PsVs in four human or monkey cell lines (Fig. 3b), which we have previously validated to have the highest susceptibility to SARS-CoV-2 infection^6,12,36^. It has been reported previously that entry into most (including Huh7, A549, and Vero E6) if not all of the cell lines tested depends on CTSL activity^12,37^. All these cell lines can be infected with WT PsV. However, after the CTSL cleavage sites were mutated to glycine in CS mutant, these PsVs gradually lost most of their infection ability in all four cell lines. For CS-1M + 2 M PsV with both CTSL cleavage sites mutated, the viral infectivity was reduced 100–700-fold compared with that of WT PsV (Fig. 3b), showing that the two CTSL cleavage sites, especially CS-2, are essential for SARS-CoV-2 infection.

Moreover, we also examined the infectivity in three 293 T/ACE2 cell lines with stable overexpression of *CTSL*, *TMPRSS2,* or *FURIN* (Fig. 3b). We found that the reduction in infectivity of CS mutant PsVs could not be fully rescued by overexpression of *CTSL, TMPRSS2,* or *FURIN*, indicating that the function of CTSL could not be fully substituted by TMPRSS2 or furin. Similar results were also observed with different PsV concentrations (Supplementary Fig. S9c).

The fusogenic activity of SARS-CoV-2 S is a character of SARS-CoV-2 infection, and the presence of syncytia in COVID-19 patient lung tissue indicates that this activity plays a role in the pathological process^38^. Syncytium formation was quantified with an ESR1-ERE transactivation system (Fig. 3c–e; Supplementary Fig. S10a, b) and observed by bright-field microscopy in our study (Fig. 3f). With the similar expression levels of S protein in transfected cells (Supplementary Fig. S10c), compared to the control group, the CS-1M group exhibited significantly reduced syncytium formation (Fig. 3e, f), while syncytium formation was inhibited even more severely in the CS-2M group, and almost no syncytia were observed in the CS-1M + 2 M group (Fig. 3e, f). More importantly, the inhibitory effects of these mutations were not rescued by the addition of CTSL into the system (Fig. 3e, f), confirming the promotive effect of CTSL on syncytium formation, as previously shown^12^, was due to S protein cleavage at CS-1 and CS-2 sites. Taken together, our findings proved that CTSL cleavage at CS-1 and CS-2 sites is essential for SARS-CoV-2 infection.

### CTSL cleavage promotes SARS-CoV-2 infection independent of furin

As the CS-2 region is very close to the furin cleavage site, we next investigated the relationship between CTSL cleavage and furin cleavage by using PsV infection assays in human Huh7 cell line^6,36,39^ under *CTSL* overexpression or knockdown conditions (Supplementary Fig. S11). For WT PsV, overexpression of the *CTSL* gene markedly increased its infection efficiency, while knockdown of this gene significantly reduced the infection efficiency (Fig. 3g), similar to our previous finding^12^. This result indicated that CTSL plays a crucial role in the SARS-CoV-2 infection process. Furthermore, for the FM-mutants PsVs, overexpression or knockdown of the *CTSL* gene also significantly affected the infection levels in a dose-dependent manner (Fig. 3g), similar to the effects on WT PsV. These results suggested that CTSL-enhanced SARS-CoV-2 viral entry is independent of furin cleavage.

The furin cleavage site has been reported required for SARS-CoV-2 S driven cell–cell fusion^13^. Here, we used the same cell fusion system (Fig. 3d) to evaluate the effect of CTSL on FM-mutants-mediated syncytium formation. FM-ARAA PsV induced a low level of syncytium formation similar to that of the control group (Fig. 3h, i). However, after CTSL was added to the mutant group, syncytium formation increased significantly, and this rescue effect exhibited a dose dependency with the concentration of CTSL (Fig. 3h, i). This result suggested that CTSL cleavage is an important furin-independent factor to induce SARS-CoV-2 S driven cell–cell fusion.

### CTSL inhibitors prevent SARS-CoV-2 infection

Since CTSL plays a key role in mediating SARS-CoV-2 infection, we selected six compounds (K777, cathepsin inhibitor 1, E64d, Z-FY-CHO, MDL-28170, and oxocarbazate) that can inhibit CTSL activity by over 90% with a concentration ranging from 30 nM to 50 μM, respectively (Supplementary Fig. S12), and evaluated their effects on SARS-CoV-2 infection.

**Fig. 4:**
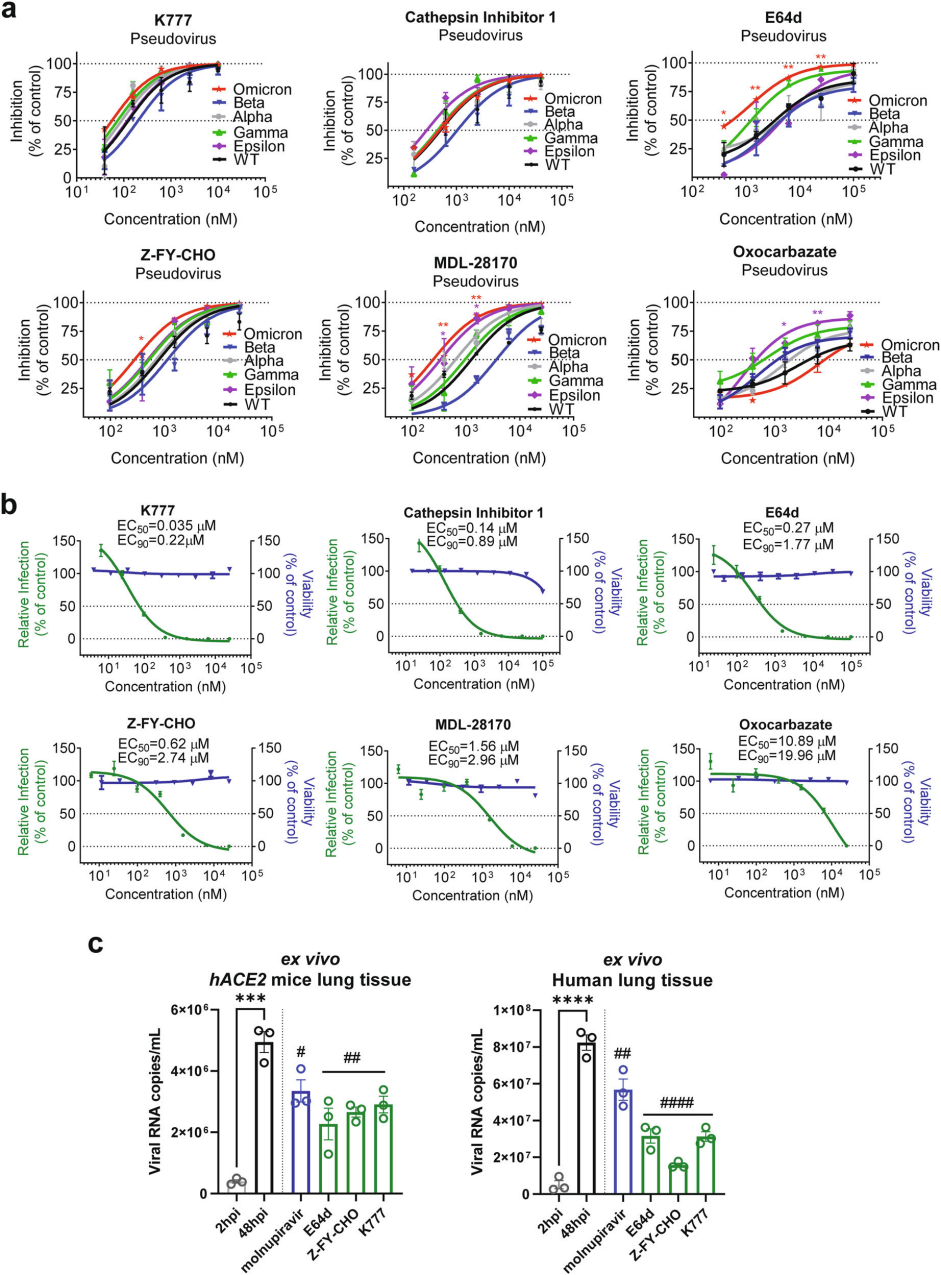
CTSL inhibitors prevent infection with SARS-CoV-2 and mutant variant PsVs in vitro. **a** Vero E6 cells were pretreated with increasing concentrations of each compound for 16 h and were then infected with different SARS-CoV-2 variant PsVs as indicted. At 24 hpi, infectivity was measured by a luciferase assay. The data were normalized to the average value in vehicle-treated cells and are shown as inhibition rates (*n* = 3). Statistical significance was assessed between the indicated variant and WT PsV by two-way ANOVA with Dunnett’s post-hoc test. The data are presented as the means ± SEM. *P < 0.05, **P < 0.01. **b** Vero E6 cells were pretreated with increasing concentrations of each compound for 16 h and were then infected with SARS-CoV-2 at an MOI of 0.01. At 24 h post-infection, viral RNA copies in supernatants were quantified by RT-qPCR. The data were normalized to the average value in vehicle-treated cells and are shown as relative infection percentages. The EC_50_ values for each compound are indicated. Cell viability was evaluated with a CCK kit (TransGen Biotech) (*n* = 3). **c** Ex vivo lung tissues from *hACE2* mice or a human donor were infected with SARS-CoV-2 with an inoculum of 1 × 106 PFU/mL for 2 h. Then the inoculum was removed and changed with medium with indicated compounds (10 μM for molnupiravir, 4 μM for E64d, 5 μM for Z-FY-CHO, and 0.4 μM for K777) for another 48 h. Tissues were harvested (without adding compounds) at 2 hpi or 48 hpi to determine the viral growth ability. Tissues were harvested at 48 hpi for quantification of viral RNA (*n* = 3). Statistical significance was assessed between 2 hpi and 48 hpi by unpaired two-tailed Student’s *t*-test. *P < 0.05, **P < 0.01, ***P < 0.001, ****P < 0.0001. Statistical significance was assessed between the indicated drug and 48 hpi by one-way ANOVA with Tukey’s post-hoc test. ^#^*P* < 0.05, ^##^*P* < 0.01, ^###^*P* < 0.001, ^####^*P* < 0.0001. The data are presented as the means ± SEM.

Firstly, we examined the antiviral effects of the six compounds against variants of SARS-CoV-2 (including the recently emerged Omicron variant) using the PsV infection assays (Fig. 4a). As we expected, all six compounds can efficiently inhibit infection of all mutant PsVs variants. Among them, E64d and MDL-28170 showed higher inhibitory efficiency against Omicron variant, while oxocarbazate was more effective against Epsilon variant. It seems that CTSL inhibitors are most likely resistant to mutational escape of SARS-CoV-2, including Omicron and Beta variants, against which the effects of most current vaccines are reduced^9,10,40^. Neither of the compounds inhibited control vesicle stomatitis virus (VSV) PsV virus infection (Supplementary Fig. S13), indicating that the inhibitory effects of CTSL inhibitors depend on SARS-CoV-2 S protein.

Secondly, in the cellular experiments using live SARS-CoV-2 virus, all six compounds efficiently blocked SARS-CoV-2 infection at non-toxic doses, with half-maximal effective concentration (EC_50_) values ranging from 35 nM to 10.9 μM (Fig. 4b). All compounds decreased the viral RNA load in Vero E6 cells by ~103- to 104-fold at the maximal non-toxic dose. This finding further confirmed the critical role of CTSL cleavage in SARS-CoV-2 infection. To be noted, we confirmed that all drugs lack potent inhibitory activity on the viral PLpro and 3CLpro (Supplementary Fig. S14), and also on human furin and TMPRSS2 (Supplementary Fig. S15) under the highest tested concentrations used in this infection study, indicating that the antiviral activities of these compounds are most likely due to the CTSL-specific inhibition.

Thirdly, we further validated the antiviral effects of the compounds in an ex vivo lung culture system. Lung tissues from human *ACE2* (*hACE2*)-transgenic mice or a human donor were infected with the live SARS-CoV-2 virus. First, tissues were harvested (without adding drugs) at 2 h post-infection (hpi) and 48 hpi to examine the system. The viral RNA copies from tissues harvested at 48 hpi were significantly higher than those from 2 hpi, indicating that the tissues were successfully infected (Fig. 4c). Then, lung tissues were infected and treated with the selected compounds, with molnupiravir as a control. Three CTSL inhibitors (E64d, Z-FY-CHO, and K777) were chosen for their lower EC_50_ and safety for in vivo studies^12^. All compounds were used in the dose of double EC_90_ concentrations assessed from Vero E6 cells^41^ (10 μM for molnupiravir^42^, 4 μM for E64d, 5 μM for Z-FY-CHO, and 0.4 μM for K777). After 48 h, tissues were harvested and the viral RNA copies were quantified. All CTSL inhibitors potently antagonized viral replication in human and mouse lung tissues, even with stronger effects than molnupiravir (Fig. 4c).

**Fig. 5:**
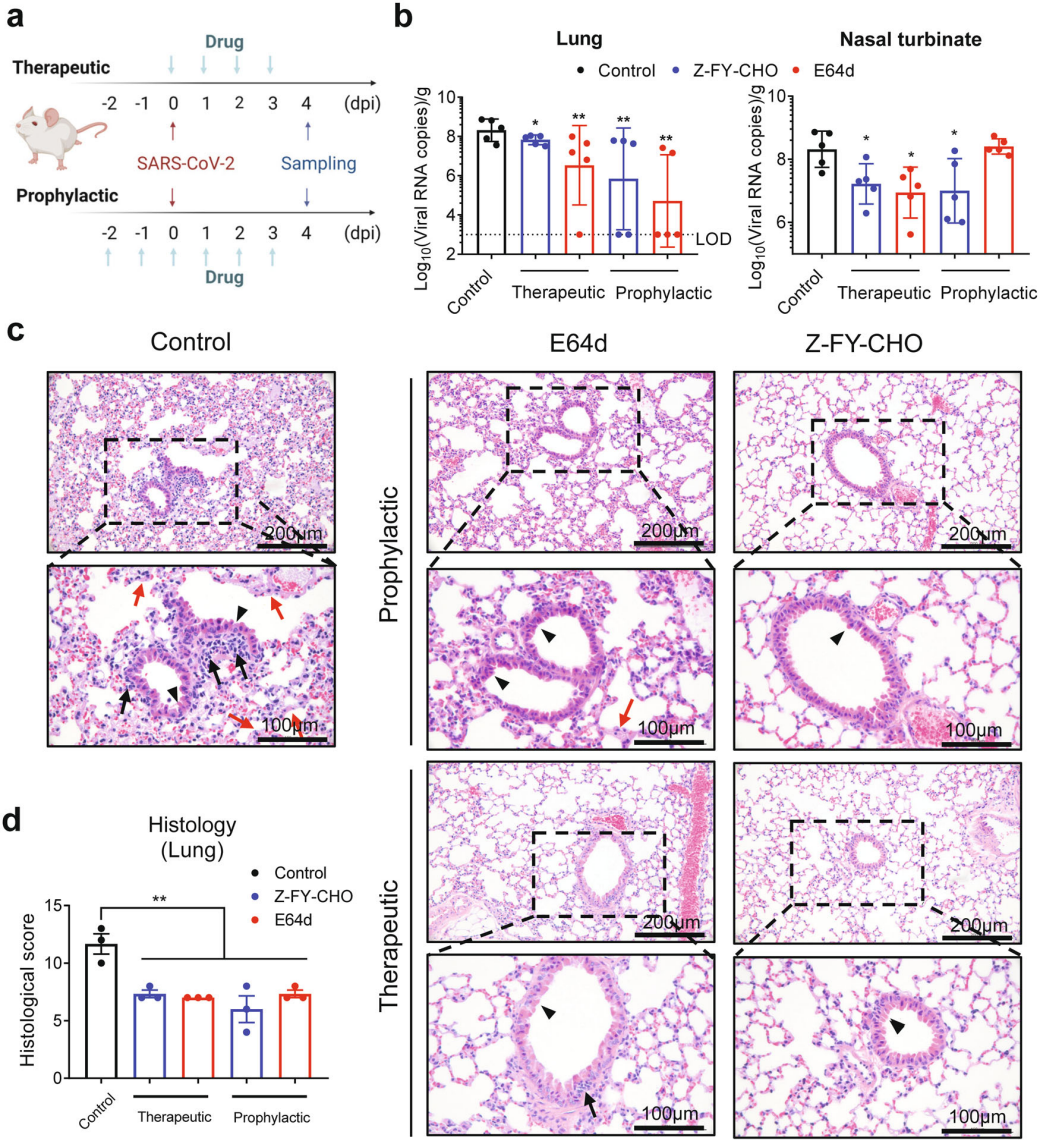
CTSL inhibitors prevent SARS-CoV-2 infection in vivo. **a** E64d and Z-FY-CHO were administered intraperitoneally at 2 days before infection to 3 dpi as prophylactic treatment, and mice were challenged with 106 PFU at 0 dpi; the two drugs were administered therapeutically at 0–3 dpi. Tissue samples were collected at 4 dpi. **b** Viral RNA copies in mouse lung and nasal turbinate tissues (*n* = 5 mice/group). The dotted line indicates the lower limit of detection (LOD). Statistical significance was assessed between the indicated group and control group by one-way ANOVA with Tukey’s post-hoc test. **c** Representative images from histological analysis of lungs from SARS-CoV-2-infected *hACE2* mice at 4 dpi. Magnified views of the boxed regions in each image are shown below the corresponding image. The black arrows indicate inflammatory cell infiltration, the black arrowheads indicate bronchiolar epithelial cell degeneration, and the red arrows indicate alveolar septal thickening. The scale bars are indicated in the figures. **d** Semiquantitative histological scoring of each lung tissue was performed by grading the severity of bronchiolar epithelial cell damage (0–10), alveolar damage (0–10) and inflammatory cell infiltration in blood vessels and bronchioles (0–10) and summing these scores to calculate the total score. Normal = 0, indeterminate = 1–2, mild = 3–4, moderate = 5–7, severe = 8–10. (*n* = 3) Statistical significance was assessed between the indicated group and control group by one-way ANOVA with Tukey’s post-hoc test.

Finally, we investigated the in vivo antiviral effects of CTSL inhibitors in *hACE2* mice, which are susceptible to SARS-CoV-2 infection^43^. We selected two compounds, E64d and Z-FY-CHO, which have been validated to be safe for in vivo studies^12,44^. E64d and Z-FY-CHO were administered in prophylactic and therapeutic regimens, and mice were treated at appropriate doses according to previous studies^12,44^. Each mouse was infected with 106 plaque-forming units (PFU) SARS-CoV-2 at 0 days post-infection (dpi) by intranasal instillation. Tissue samples were collected at 4 dpi (Fig. 5a), when the viral load peaked, and exhibited obvious histopathological changes. Both compounds significantly decreased the number of viral RNA copies in lung tissues by ~1–4 log10, with undetectable (below the lower limit of detection, LOD) viral loads in lung tissues from two mice treated with Z-FY-CHO and four mice treated with E64d (Fig. 5b). Both drugs also significantly decreased the viral load in the nasal turbinate (Fig. 5b). Histological analysis of the lungs showed that vehicle-treated mice exhibited moderate pathological changes, as evidenced by the large areas of alveolar septal thickening, inflammatory cell infiltration and bronchiolar epithelial cell degeneration. In contrast, the lungs of E64d- and Z-FY-CHO-treated mice exhibited improved morphology and less infiltration (Fig. 5c, d). Taken together, these results indicated that CTSL inhibitors efficiently block SARS-CoV-2 infection both in vitro and in vivo and are resistant to viral mutational escape.

## Discussion

In this study, we identified two specific cleavage sites of CTSL in the SARS-CoV-2 S protein, named CS-1 and CS-2. Both sites are located in the flexible loop regions within S1 subunit, while CS-1 is in the NTD and CS-2 is in the CTD near the S1/S2 furin cleavage site. Both sites are highly conserved among all SARS-CoV-2 variants, including the most recently emerging variants. Our structural studies proved that CTSL cleavage can greatly enhance S protein dynamics with an increased proportion of activated states ready for receptor-binding and membrane fusion. The viral infection assay using the PsV system proved that CTSL cleavage, especially cleavage at CS-2, is an essential step in SARS-CoV-2 infection. Furthermore, CTSL-specific inhibitors not only blocked the infection of PsV/live virus in cells but also reduced the infection rate of live virus significantly ex vivo and in vivo. Our results suggested CTSL cleavage is an important process during SARS-CoV-2 infection.

**Fig. 6:**
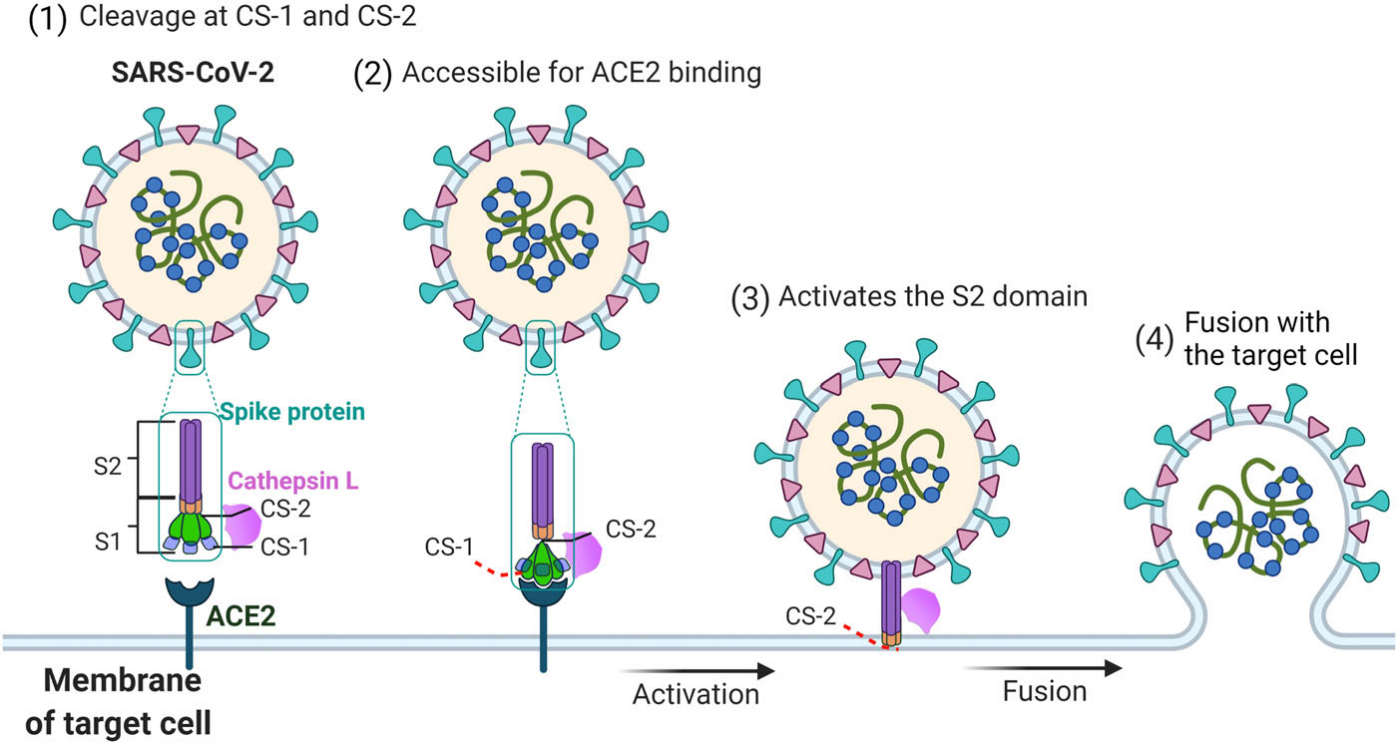
Proposed mechanism by which CTSL promotes SARS-CoV-2 infection. SARS-CoV-2 S contains S1 and S2 subunits. CS-1 is located in the NTD of the S1 subunit, and CS-2 is located near the S1/S2 site. CTSL cleaves the SARS-CoV-2 S protein: (1) By binding with the S protein on the surface of SARS-CoV-2, CTSL cleaves S at CS-1 and CS-2 sites. (2) The cleavage increases the dynamics of the RBD and makes it accessible to ACE2 for binding. (3) CTSL cleaves CS-2 site to separate S1 and S2 subunits to expose the S2 subunit for membrane fusion. (4) The virus fuses with the membrane of target cell, and the viral genetic material is released into the host cell.

Based on our structural and functional study, we proposed a possible model for the CTSL-mediated SARS-CoV-2 infection process (Fig. 6). When the viral S protein encounters activated CTSL, the cleavage occurs at both CS-1 and CS-2 sites on the S protein. Then dynamics of the RBD and NTD are increased to facilitate the activation of S protein and promote ACE2 binding. Next, the cleavage at CS-2 site separates S1 and S2 domains to expose S2 ready for membrane fusion and then complete the infection process. Considering the localization and activity characteristics of CTSL, these cleavage reactions are most likely to occur in the endosomes.

Previous studies indicated that SARS-CoV-1, MERS-CoV, and SARS-CoV-2 were all capable of using both endosomal and membrane fusion routes to enter the cell^13,45^. TMPRSS2 appears to be one of the major proteases for priming S for entry via the plasma membrane, whereas CTSL performs the priming function during entry through the endosome in vitro^13,45^. In support of this view, endosomal acidification inhibitors have been shown to effectively block the entry of SARS-CoV-1 and SARS-CoV-2 by preventing the activation of CTSL^29,45^. Cell culture studies showed that TMPRSS2 was more significantly associated with the entry of SARS-CoV-2 into Calu-3 cells^13^, while the endosomal entry route might be dominant for SARS-CoV-2 into Vero cells^46^. Therefore, the flexibility of protease usage and entry pathway seems to be a consistent strategy for coronavirus use, and in some cases, its role depends on cell type. In patients with SARS-CoV-2 infection, the most susceptible organ is the lung, which contains many kinds of cell type. In this study, we further validated the antiviral effects of the CTSL inhibitors in human lung tissue ex vivo.

In addition, SARS-CoV-2 attachment cofactors can facilitate SARS-CoV-2 S binding to ACE2. Subsequently, the cleavage of cell surface proteases can lead to cell membrane fusion^23^. Our study indicates that CTSL can attach S protein at CS-1 site, implying that may CTSL serve as SARS-CoV-2 attachment cofactor to facilitate SARS-CoV-2 S binding to ACE2.

It is well known that furin and TMPRSS2 are important human enzymes that are employed by SARS-CoV-2 during the infection process. The expression of TMPRSS2 has been confirmed within COVID-19 relevant lung cell types^19^ and its role in mediating SARS-CoV-2 infection has been proved^13^. TMPRSS2 also has been reported to mediate ACE2 activation in SARS-CoV-1 infection^47^. Here, we further identified that SARS-CoV-2 can utilize human CTSL to assist infection independent of furin. Therefore, all these human enzymes, including CTSL, TMPRSS2, and furin, could be involved in the viral infection process, and are most likely to play complementary or compensatory roles in different human tissues. Drugs targeting these human enzymes should be paid more attention to.

Moreover, for the drugs that do not target the virus directly, they would be less easily escaped by the SARS-CoV-2 variants. As expected, we found that the CTSL-specific inhibitors can nearly completely prevent infection of SARS-CoV-2 variant PsVs and reduce infection of live SARS-CoV-2 by 103–104-fold in Vero E6 cells. More importantly, CTSL-specific inhibitors can effectively prevent SARS-CoV-2 infection in *hACE2-*transgenic mice. These results have provided important evidence for the development of next-generation broad-spectrum drugs against SARS-CoV-2.

In our study, we found that both CTSL cleavage sites (CS-1 and CS-2) possess extra high sequence conservations among SARS-CoV-2 variants, indicating their importance for the viral life cycle. It may be inferred that those two cleavage sites cannot be easily mutated in the live SARS-CoV-2 variants, or that the variant with mutations in those two sites might have weak infectivity, which is indicated in our experiments that the mutations of these two sites, especially CS-2 site, largely impaired the infectivity of the virus. Therefore, the development of specific drugs or vaccines targeting CS-1 and CS-2 sites would be a new strategy to prevent SARS-CoV-2. If these sites are occupied by drugs or antibodies which can block CTSL cleavage, the viral infection could be effectively inhibited. This approach would be much safer than the strategy of using compounds to inhibit the activity of multiple human proteases.

In conclusion, the high mutation rate of SARS-CoV-2 often leads to viral escape from neutralizing antibodies or vaccines. An understanding of the precise mechanisms underlying SARS-CoV-2 infection is urgently needed to facilitate the development of broad-spectrum antiviral drugs or vaccines with increased resistance to viral mutations. Our study shows that CTSL and its new cleavage sites at the SARS-CoV-2 S protein are the crucial new targets for the future development of anti-SARS-CoV-2 therapy.

## Materials and methods

### Protein expression and purification

The S_2P_ protein was purified as previously reported^24^. Briefly, the prefusion S ectodomain, the mammalian codon-optimized gene coding SARS-CoV-2 (Wuhan-Hu-1 strain, GenBank ID: MN908947.3) S glycoprotein ectodomain (residues M1–Q1208) with proline substitutions at K986 and V987, a “GSAS” substitution at the furin cleavage site (R682 to R685), a C-terminal thrombin tag, a T4 fibritin trimerization motif and a 6× HisTag was synthesized and cloned. To express 4-Gly-mutant S protein, residues W258, T259, V635, and Y636 were substituted into glycine. These expression vectors were used to transiently transfect Expi293 cells and purified by using cOmpleteHis-Tag Purification Resin to capture the target protein. The protein was then subjected to additional purification by size-exclusion chromatography using Superdex 200 10/300 GL column (GE Healthcare) in PBS.

The S_R_ protein with only an arginine left at the furin cleavage site (P681–R685) was constructed, expressed, and purified as previously described^30^. Briefly, 300 μg of plasmid was incubated with 810 μg of polyethylenimine for 10 min, and then transfected into 300 mL of 293 F cells at 2.7 million/mL. The transfected cells were cultured at 33 °C. After 4 days, cell culture supernatant was collected and then loaded onto 5 mL Hitrap Talon Cobalt column. The column was washed with 100 mL buffer A (25 mM phosphate pH 8.0, 300 mM NaCl, 5 mM imidazole) and eluted with 100 mL 0–100% linear gradient of buffer B (25 mM phosphate pH 8.0, 300 mM NaCl, 200 mM imidazole). After that, the protein was concentrated and buffer exchanged into PBS by a 100 kDa MWCO ultra centrifugal filter (Millipore).

### CTSL treatment

Recombinant CTSL (Novoprotein) was used to cleave SARS-CoV-2 S_2P_ protein. Purified SARS-CoV-2 S protein was incubated with CTSL (2–8 μg/mL as indicated and 8 μg/mL for cryo-EM sample preparation) in the presence of 100 mM NaAC, 1 mM EDTA and 1 mM dithiothreitol (DTT), pH 5.5 for 24 h at 25 °C. CTSL was preactivated at 30 °C for 1 min before use. The resulting protein was then subjected to sodium dodecyl sulfate–polyacrylamide gel electrophoresis (SDS-PAGE) and stained by coomassie blue or silver staining methods as indicated. For cryo-EM sample preparation, the S_R_ protein was firstly cleaved by TEV enzyme in a mass ratio at 10:1 for 16 h at 25 °C, then treated with CTSL in pH 5.5 for 24 h at 25 °C.

### Sequence analysis

Sequence alignments were performed using the CLUSTALW online tool (https://www.genome.jp/tools-bin/clustalw). The representative variant sequence of each SARS-CoV-2 PANGO lineage was obtained from Global Initiative on Sharing All Influenza Data (GISAID) database: B.1.1.529 (GISAID: EPI_ISL_6900143), B.1.617 (GISAID: EPI_ISL_1544002), P.1 (GISAID: EPI_ISL_1164984), B.1.351 (GISAID: EPI_ISL_935042), B.1.1.7 (GISAID: EPI_ISL_1257795), B.1.621 (GISAID: EPI_ISL_6864938), C.37 (GISAID: EPI_ISL_6794513), AY.4.2 (GISAID: EPI_ISL_6905104), C.36 (GISAID: EPI_ISL_6866297), C.1.2 (GISAID: EPI_ISL_6906407), B.1.427 (GISAID: EPI_ISL_1221570), B.1.1.318 (GISAID: EPI_ISL_6661876), B.1.429 (GISAID: EPI_ISL_1160035), B.1.640 (GISAID: EPI_ISL_6887009), B.1.525 (GISAID: EPI_ISL_6903493), P.3 (GISAID: EPI_ISL_6756120), B.1.617.1 (GISAID: EPI_ISL_6877952). In addition, the following sequences information were obtained from the National Center for Biotechnology Information (NCBI) database: SARS-CoV BJ01 (GenBank: AAP30030.1), MERS-CoV (GenBank: QBM11748.1), HCoV NL63 (GenBank: APF29063.1), HcoV 229E (GenBank: AWH62679.1), HcoV OC43 (GenBank: AIX10763.1), HcoV HKU1 (GenBank: AMN88694.1).

### N-terminal Edman sequencing and LC-MS/MS analysis

CTSL-treated SARS-CoV-2 S protein was resolved on an SDS-PAGE gel. The protein was transferred to PVDF membrane before Edman sequencing. Each protein band was excised individually. The sequence of the first 10 amino acids for each band was determined by Edman sequencing using ABI Procise-cLC machine. LC-MS/MS analysis of excised bands was performed at Laboratory of Proteomics, Institute of Biophysics, Chinese Academy of Sciences.

### Cryo-EM sample preparation and data collection

For each grid, 3 μL of purified protein solution of CTSL-treated or untreated SARS-CoV-2 S proteins were applied to newly glow-discharged holy carbon film grids (R1.2/1.3, 200 meshes, Au, Quantifoil, Germany) or holy Ni-Ti film grids^48^ (R1.2/1.3, 300 meshes, Au, Zhenjiang Lehua Electronic Technology Co., LTD, China). Then the grid was blotted and vitrified by plunge freezing into liquid ethane using Vitrobot Mark IV (Thermo Fisher Scientific, USA) at 4 °C and 100% humidity. All movies were collected on a Titan Krios G2 TEM (Thermo Fisher Scientific, USA) operated at 300 KV on EF-TEM mode with nominal magnification set to be ×105,000, resulting in a calibrated physical pixel size of 1.36 Å on a specimen level. The total dose was set to be 60 e−/Å^2^, with an exposure time of 15 s. The movies were acquired by Gatan K2 Summit DDD detector (Gatan Company, USA) equipped with a GIF Quantum energy filter, with a silt width of 20 eV, operated in super-resolution mode, resulting in a pixel size of 0.68 Å for output super-resolution movies. SerialEM^49^ with in-house scripts were used for data collection^50^, defocus values for either samples were set between −1.0 and −2.0 μm.

### Image processing

Image processing steps were performed using RELION^51,52^ and cryoSPARC^53^. The output super-resolution movies were first subjected to motion correction using MotionCor2^54^, with the binning level of 2 in Fourier space, and dose weighting was also performed during the process. Contrast transfer function (CTF) parameters estimation were performed using Gctf^55^. Gautomatch (https://www.mrc-lmb.cam.ac.uk/kzhang/Gautomatch/) was used to pick all particles from raw micrographs. Then good particles were extracted and sorted by 2D classification. After 2D classification, one round of 3D classification was performed to investigate different populations of S proteins. To prevent any model bias, the previously reported cryo-EM structure of SARS-CoV-2 S (Protein Data Bank, PDB entry 6VXX) lowpass filtered to 60 Å was used as the initial reference and no additional symmetry was imposed throughout the initial image processing pipeline. Then the well-aligned classes with clear secondary structure features were selected separately for subsequent image processing, including auto-refinement, Bayesian polishing, CTF refinement, and no-alignment 3D classification. All these steps were performed in RELION. For the final round of refinement, the non-uniform (NU) refinement in cryoSPARC was performed to refine the final maps to a higher resolution.

### Model building and refinement

All the cryo-EM maps are improved by density modification procedures before model building^56^. To build the atomic model of CTSL-treated or untreated SARS-CoV-2 S structures, the reported cryo-EM structure of SARS-CoV-2 S (PDB entry 6VXX & 6VYB)^22^ was used as an initial model. We were able to trace most regions with side chains using COOT^57^. The built model was further refined in real space using Phenix^58^. All figures were created by Pymol^59^, UCSF Chimera^60^, and UCSF ChimeraX^61^. The parameters for data collection and structure determination are summarized in Supplementary Table S2. The overall model building quality is shown in Supplementary Videos S1–S4.

### BLI

The BLI assay was performed at 30 °C on the Octet RED96 System (ForteBio) with 100 mM NaAC, 1 mM EDTA, and 1 mM DTT, pH 5.5 and 0.02% Tween-20 as running buffer. Purified human IgG Fc-hACE2 (Novoprotein) was loaded for 300 s onto Anti-Human IgG Fc Capture (AHC) biosensors (ForteBio). To measure the binding affinity between ACE2 and S variants, the biosensors were then incubated with various concentrations of the purified WT S_2P_ and CS mutant (CS-1M + 2 M) S_2P_ proteins for 90 s, followed by 180 s of dissociation. Both WT and CS mutant S_2P_ proteins have proline substitutions at K986 and V987 and a “GSAS” substitution at the furin cleavage site. For the CTSL cleavage activation assay, the biosensors were incubated for 180 s with various concentrations of S_R_ before and after CTSL treatment, followed by 200 s of dissociation. The data were analyzed using the software supplied by the manufacturer.

### Cell culture

The Huh7 (*Homo sapiens*, liver), 293T-hACE2 (293 T cells stably expressed, *Homo sapiens*, embryonic kidney), Vero (*Cercopithecus aethiops*, kidney), VeroE6 (*Cercopithecus aethiops*, kidney), and LLC-MK2 (*Macaca mulatta*, kidney) cells were maintained in high glucose Dulbecco’s modified Eagle’s medium (DMEM) (Sigma–Aldrich, St. Louis, MO, USA) supplemented with 10% fetal bovine serum (FBS, Gibco, Carlsbad, CA), 100 units/mL Penicillin-Streptomycin (Gibco). All the cells were maintained at 37 °C in a humidified atmosphere containing 95% air and 5% CO_2_.

### Experimental mice

The study used human *ACE2*-transgenic mice, a mouse model expressing *hACE2* generated by using clustered regularly interspaced short palindromic repeats/CRISPR associated protein 9 (CRISPR/Cas9) knock-in technology as previously reported^43^. The *hACE2* mice used in this manuscript were 17-week-old female C57BL/6 mice, with the body weight between 22 g. All animal protocols were approved by the Ethical Review Committee at the Institute of Zoology, Capital Medical University, China.

### SARS-CoV-2-S plasmids and site-directed mutagenesis

SARS-CoV-2-S plasmid was constructed by inserted the codon-optimized S gene of SARS-CoV-2 (GenBank: MN_908947) into pcDNA3.1 as previously described^6^. For the site-directed mutagenesis, 15–20 nucleotides before and after the target mutation site were selected as forward primers, while the reverse complementary sequences were selected as reverse primers. Following site-directed mutagenesis PCR, the template chain was digested using DpnI restriction endonuclease (NEB, USA). The PCR product was transformed into *E. coli* DH5α competent cells; single clones were selected and then sequenced.

### Production and quantification of PsVs

PsVs incorporated with S protein from either SARS-CoV-1, SARS-CoV-2, or mutants were constructed using a procedure described by us previously. For this VSV-based PsV system, the backbone was provided by VSV-G pseudotyped virus (G*ΔG-VSV) that packages expression cassettes for firefly luciferase instead of VSV-G in the VSV genome. For quantification of PsV, viral RNA was extracted by using the QIAamp Viral RNA Mini Kit (Cat. No. 52906, QIAGEN), and the reverse transcription was performed with RevertAidTM First Strand cDNA Synthesis Kit (Fermentas K1622) according to the manufacturer. The real-time qPCR was then performed on the LightCycler® 96 Real-Time PCR System (Roche) using SYBR Green I Master Mix reagent (Roche). The P protein gene of VSV virus was quantified and the viral copy number calculated accordingly. The primers were: forward-TCTCGTCTGGATCAGGCGG; reverse-TGCTCTTCCACTCCATCCTCTTGG. All PsVs were normalized to the same amount as previously described^6^.

### Western blot analysis

7 mL of SARS-CoV-2 PsVs with a titre of 1.86 × 105 50% tissue culture infectious dose/mL (TCID_50_/mL) were pelleted through a 25% sucrose cushion by ultra-centrifugation at 100,000 × *g* for 3 h. The layer of supernatant was discarded. The pellet was collected and subjected to western blot analysis as previously described^62^, and detected by anti-S1 mice serum, VSV-M antibody (KeraFast, Cat. No. EB0011), anti-GAPDH antibody (Sigma), and anti-His antibody (Sigma).

### PsV infection assay

Before infection, the 96-well plates were seeded with cells adjusted to 2 × 105 cells/mL. Then, 100 μL of the normalized PsV with indicated dilution fold was added to wells in 96-well cell culture plate. After 24 h incubation at 37 °C, the activities of firefly luciferase were measured on cell lysates using luciferase substrate (PerkinElmer, BRITELITE PLUS 100 mL KIT, Cat. No. 6066761) following the manufacturer’s instructions. Luciferase activity was measured using a luminometer (Promega).

### Overexpression and knockdown of CTSL

Overexpression and knockdown of *CTSL* gene in Huh7 cells were described previously^12^. Briefly, for *CTSL* knockdown, Huh7 cells were plated in 48-well plates, and transfected with indicated concentrations of siRNAs against homo *CTS*L mRNA (si-CTSL) or 50 nM negative control siRNA (scramble) using Lipofectamine 3000 reagent (Thermo Fisher Scientific). For *CTSL* overexpression, Huh7 cells were plated in 48-well plates and transfected with indicated concentrations of human CTSL expression plasmid (GenBank: NM_001912, Vigenebio) or 0.2 μg control plasmid. The overexpression and knockdown efficiencies were validated in both mRNA and protein levels by us previously^12^. Twenty-four hours post-transfection, the medium was replaced with a fresh medium. Then the cells were infected with SARS-CoV-2 or mutant PsV (1.3 × 104 TCID_50_/mL) and cultured for another 24 h before firefly luciferase activity analysis. siRNA sequences were provided in Supplementary Table S3.

### Syncytium-formation assay

Syncytium-formation assay was performed as previously described by us^12^. Briefly, Huh7 cells were seeded in 24-well plates and transfected with SARS-CoV-2-S or mutant protein expression plasmids using Lipofectamine 3000 reagent (Thermo Fisher Scientific). The transfection solutions were changed to a standard culture medium 4–6 h post-transfection and cells incubated for an additional 6–8 h. Next, cells were treated in the absence (PBS, pH = 5.8) or presence of 2 or 4 μg/mL CTSL (Novoprotein) (in PBS, pH = 5.8) for 20 min at 37 °C. Then, the solutions were changed to a standard culture medium, and the cells further incubated for 10–16 h. The pictures were captured under bright-field microscopy (Olympus).

### Quantification of syncytium formation

Huh7 target cells were seeded in 48-well plate at 10%–20% confluency and transfected with ESR1 expression plasmid. Huh7 effector cells were seeded in a six-well dish at 50%–70% confluency and transfected with ERE-Luciferase reporter plasmid as well as expression plasmid for WT or mutant SARS-CoV-2-S or empty plasmid (1:2 ratio). At 24 h post-transfection, effector cells were detached by resuspending and added to the target cells at 1:1 ratio. Where indicated, the medium was changed by CTSL, trypsin or PBS 2 h after the mixture and the reaction was stopped after 20 min by adding 500 μL culture medium. Luciferase activity was analyzed after 20–30 h.

### Analysis of CTSL, Furin, TMPRSS2, viral 3CLpro, and PLpro activity

The protease inhibition by drugs was measured using different substrates: Ac-FR-AFC (Abcam, Cat. No. ab157769) for CTSL (Novoprotein), BOC-QAR-AMC (Absin, Cat. No. abs45133013) for TMPRSS2 (CUSABIO), MCA-AVLQSGFR-Lys (Dnp)-Lys-NH_2_ (Beyotime, Cat. No. P0315S) for 3CLpro (Novoprotein), and Z-RLRGG-AMC (TGpeptide) for PLpro (Sino Biological). Furin activity was measured using a commercial kit (AnaSpec, Cat. No. AS-72256). The protease activity was measured by a spectrophotometer (Promega) at the appropriate wavelength.

### SARS-CoV-2 virus

All experiments with infectious SARS-CoV-2 were performed in the biosafety level 4 and animal biosafety level 4 facilities in the Harbin Veterinary Research Institute (HVRI) of the Chinese Academy of Agricultural Sciences (CAAS), which is approved by the Ministry of Agriculture and Rural Affairs of China.

SARS-CoV-2 strain SARS-CoV-2/HRB25/human/2020/CHN (HRB25, GISAID: EPI_ISL_467430)^63^ was isolated from a patient in Vero E6 cells. Viral stocks were prepared in VeroE6 cells with DMEM containing 5% FBS. Viruses were harvested and the titers were determined by means of plaque assay in Vero E6 cells^64^.

### SARS-CoV-2 infection assay in vitro

Vero E6 cells were seeded in a 96-well plate and grown overnight. Then the cells were pretreated with different concentrations of each compound for 16 h and infected with SARS-CoV-2 at a MOI of 0.01. After 24 h, the cell supernatants were collected to extract viral RNA and subjected to Real-Time quantitative reverse transcription PCR (RT-qPCR) analysis. The N gene-specific primers (forward, 5′-GGGGAACTTCTCCTGCTAGAAT-3′; reverse, 5′-CAGACATTTTGCTCTCAAGCTG-3′) and probe (5′-FAM-TTGCTGCTGCTTGACAGATTTAMRA-3′) were utilized according to the information provided by the National Institute for Viral Disease Control and Prevention, China (http://nmdc.cn/nCoV). The EC_50_ values were calculated by using a dose-response model in GraphPad Prism 8.0 software. The cell viability was determined by using the cell counting kit (CCK) (TransGen Biotech) according to the manufacture.

### SARS-CoV-2 infection assay using human lung tissues and *hACE2* mice lung tissues ex vivo

The ex vivo human lung tissue culture and virus infection experiment was approved by the Institutional Review Board of the Beijing Tongren Hospital, Capital Medical University (TRECKY2021-202). The informed consent was obtained from the participant. Fresh normal human lung tissue was obtained from a patient undergoing resection of lung tumor. Fresh *hACE2* mice lung tissue was obtained from the *hACE2* mice. Lung tissue was cut into 2–3 mm^3^ cubes and subsequently infected by a SARS-CoV-2 inoculum of 1 × 106 TCID_50_/mL for 2 h at 37 °C. After inoculation, the inoculum was removed, and the specimens were washed three times with PBS. Tissue cubes were maintained in DMEM/F12 medium supplemented with different concentrations of the indicated drugs for an additional 48 h. Then the tissues were harvested and the viral RNAs copies were measured by RT-qPCR.

### SARS-CoV-2 infection assay in vivo

Prophylactic treatment was given on –2, –1, 0, 1, 2, and 3 dpi, while therapeutic regimen was given on 0, 1, 2, and 3 dpi. The mice were infected at 0 dpi with 106 PFU of SARS-CoV-2 by intranasal instillation. Lung and nasal turbinate tissues were collected at 4 dpi. The viral RNAs copies from tissues were measured by RT-qPCR. Tissue pathology of infected mice was examined by the hematoxylin and eosin (H&E) staining and immunohistochemical assays as described previously^64,65^. Semiquantitative histological scoring of each lung tissue was performed by grading the severity of bronchiolar epithelial cell damage (0–10), alveolar damage (0–10), and inflammatory cell infiltration in blood vessels and bronchioles (0–10) and summing these scores to calculate the total score. Normal = 0, indeterminate = 1–2, mild = 3–4, moderate = 5–7, severe = 8–10.

### Statistical analysis

All statistical analyses were conducted with the software GraphPad Prism version 7.0 Data are presented as means ± SEM. Statistical significance was determined using unpaired Student’s *t*-test or the Mann-Whitney *U*-test if *n* < 9, or one-way ANOVA as appropriate.

## Supplementary information

Supplementary Information

Supplementary Video S1

Supplementary Video S2

Supplementary Video S3

Supplementary Video S4

## Data Availability

The Electron Microscopy Database (EMD) accession codes of untreated S-closed state, CTSL-treated S-closed state, S-1R-weak state, S-1R-open state are EMD-32490, EMD-32491, EMD-32492, and EMD-32493, respectively. The structural models of untreated S-closed state, CTSL-treated S-closed state, S-1R-weak state, S-1R-open state were deposited to the Protein Data Bank (PDB) with accession code of 7WGV, 7WGX, 7WGY, and 7WGZ, respectively. The other data that support the findings of this study are available from the corresponding author upon reasonable request.
