# Supplementary material for: Novel cleavage sites identified in SARS-CoV-2 spike protein reveal mechanism for cathepsin L-facilitated viral infection and treatment strategies
Source: Cell Discov. 2022 Jun 6;8:53. doi: 10.1038/s41421-022-00419-w (PMC9167920; doi:10.1038/s41421-022-00419-w)
Supplement: Supplementary file 1 — Supplementary Information [file 41421_2022_419_MOESM1_ESM.pdf]

Supplementary information for

Novel cleavage sites identified in SARS-CoV-2 spike protein reveal mechanism for cathepsin L-facilitated viral infection and treatment strategies

Miao-Miao Zhao[1,*], Yun Zhu[2,*], Li Zhang[3,*], Gongxun Zhong[4,7,*], Linhua Tai[2,5], Shuo Liu[3], Guoliang Yin[2,5], Jing Lu[1], Qiong He[1], Ming-Jia Li[1], Ru-Xuan Zhao[1], Hao Wang[1], Weijin Huang[3], Changfa Fan[6], Lei Shuai[4,7], Zhiyuan Wen[4,7], Chong Wang[4], Xijun He[4,7], Qiuluan Chen[9,10], Banghui Liu[9,10], Xiaoli Xiong[9,10], Zhigao Bu[4,7]✉, Youchun Wang[3]✉, Fei Sun[2,5,8,9] ✉ and Jin-Kui Yang[1]✉

[1]Department of Medicine, Beijing Tongren Hospital, Capital Medical University, Beijing, China

[2]National Key Laboratory of Biomacromolecules, CAS Center for Excellence in Biomacromolecules, Institute of Biophysics, Chinese Academy of Sciences, Beijing, China.

[3]Division of HIV/AIDS and Sex-Transmitted Virus Vaccines, Institute for Biological Product Control, National Institutes for Food and Drug Control (NIFDC), Beijing, China

[4]State Key Laboratory of Veterinary Biotechnology, Harbin Veterinary Research Institute, Chinese Academy of Agricultural Sciences, Harbin, China

[5]University of Chinese Academy of Sciences, Beijing, China.

[6]Division of Animal Model Research, Institute for Laboratory Animal Resources, National Institutes for Food and Drug Control, Beijing, China.

[7]National High Containment Laboratory for Animal Diseases Control and Prevention, Harbin, China

[8]Center for Biological Imaging, Institute of Biophysics, Chinese Academy of Sciences, Beijing, China.

[9]Bioland Laboratory, Guangzhou, China.

[10]The State Key Laboratory of Respiratory Disease (SKLRD), Guangzhou Institutes of Biomedicine and Health, Chinese Academy of Sciences, Guangzhou, China

✉Correspondence to Jin-Kui Yang (jkyang@ccmu.edu.cn) or Fei Sun (feisun@ibp.ac.cn) or Youchun Wang (wangyc@nifdc.org.cn) or Zhigao Bu (buzhigao@caas.cn). Leading contact: Jin-Kui Yang

Supplementary information includes 15 figures,3 tables and 4 video files.

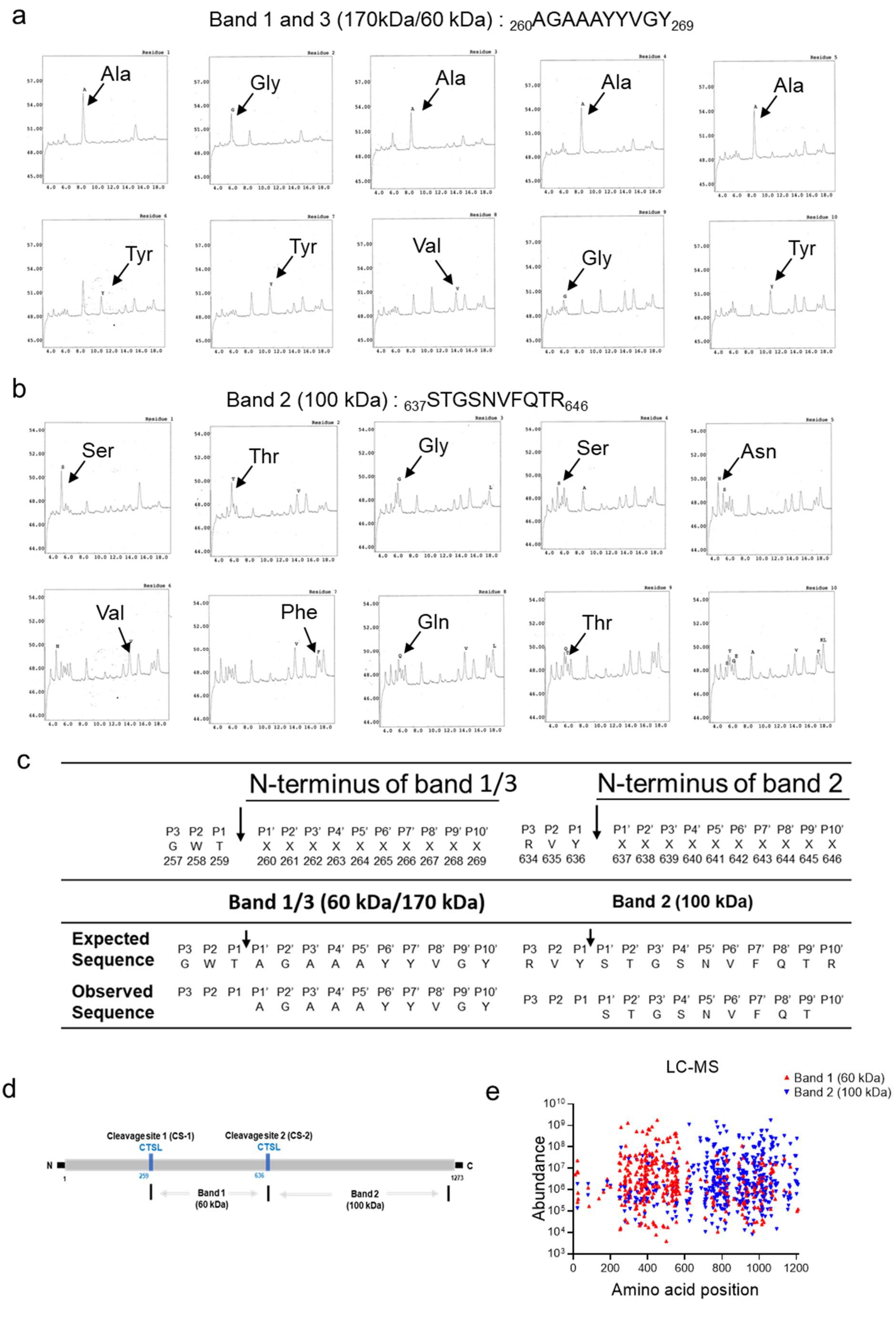

Supplementary Fig. S1. Characterization of CTSL cleavage and key residues.

a-c, Determination of the N-terminal sequence of band 1-3 using the standard Edman degradation method. The first ten amino acids in the N-terminus of band 1 and 3 **(a)** and band 2 **b,** were determined. **c,** Summary of the N-terminal residues of band 1/3 and band 2.

d, Schematic illustration of the SARS-CoV-2 S glycoprotein showing band 1 and band 2.

e, Identification of band 1 and band 2 by LC-MS. The abundance values of the identified peptides were $\log_{10}$ transformed and plotted against the mapped location in the S protein sequence.

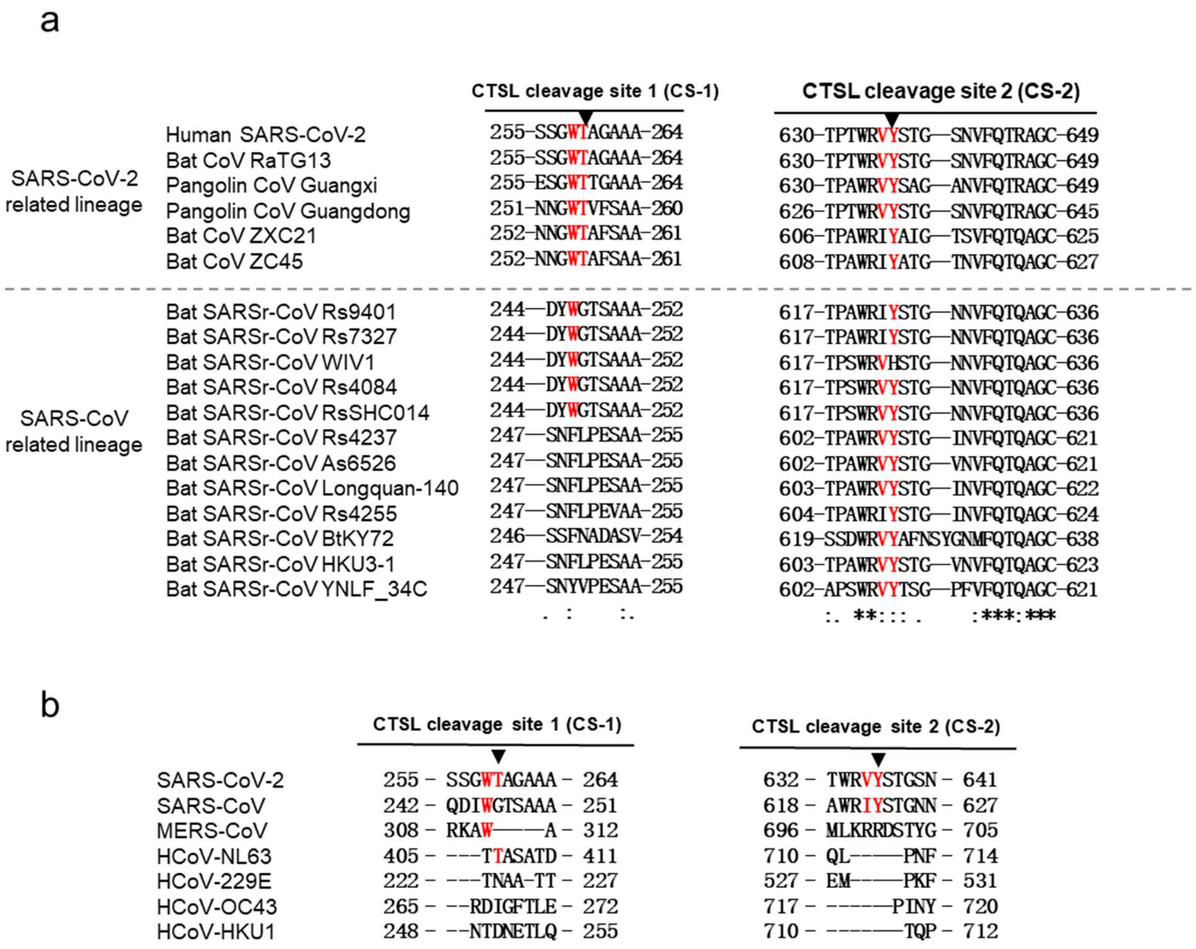

Supplementary Fig. S2. Sequence conservation of the two CTSL cleavage sites in SARS-CoV-2 S.

a-b, Amino acid sequence alignment of residues around CS-1 and CS-2 in SARS-CoV-2- and SARS-CoV-1-related coronaviruses originating from bats and pangolins **(a)** and in coronaviruses that can infect humans **(b)**. P1 and P2 residues that are similar to SARS-CoV-2 residues are highlighted in red. The symbol "*" indicates amino acid residues that are conserved among all tested sequences, and the symbols ":" and "." indicate positions with heterogeneous amino acid residues that share highly similar or similar biochemical properties.

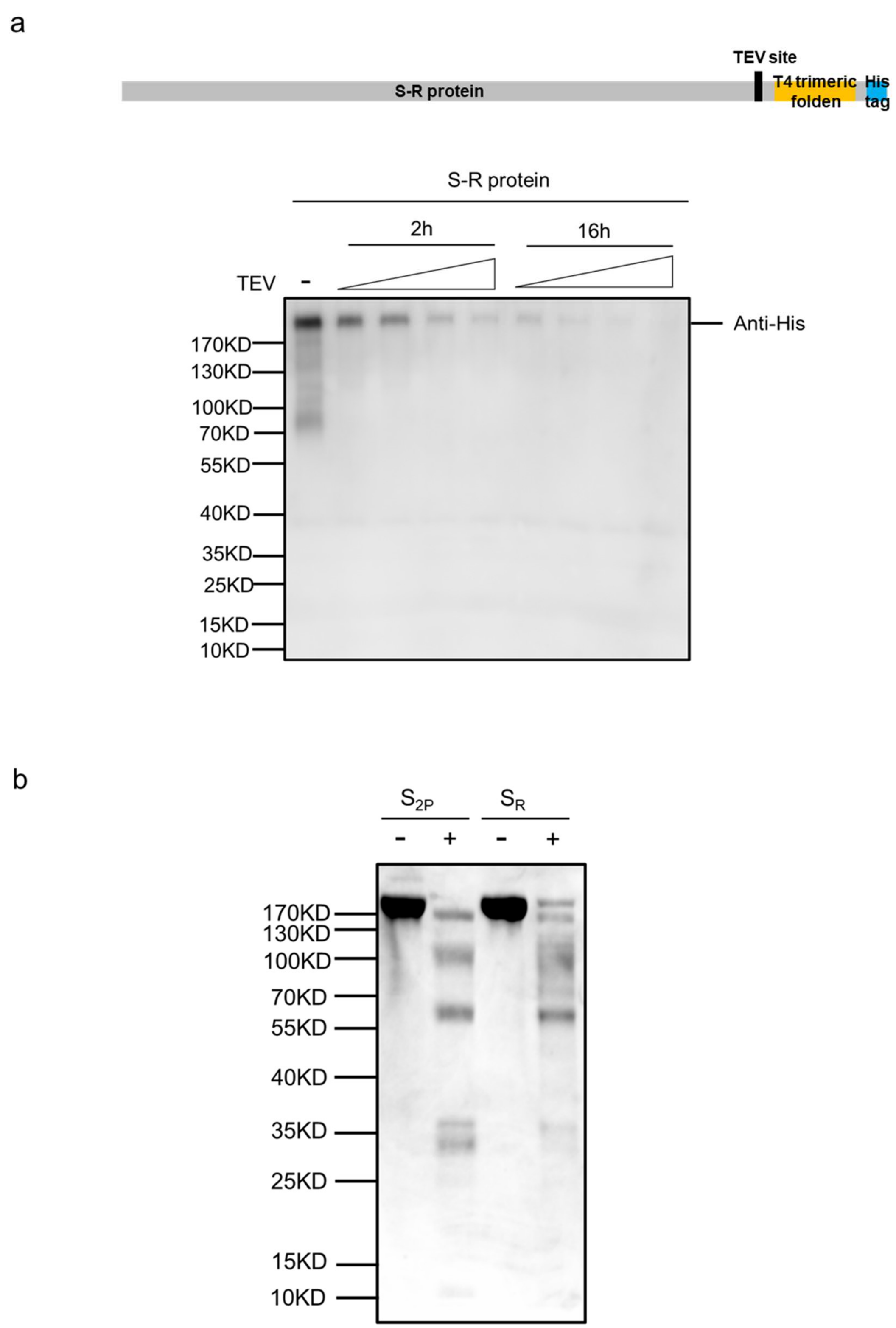

Supplementary Fig. S3. The cleavage process of S_R protein.

a, Before the cryo-EM sample preparation, the T4 trimeric foldon on the C-terminal of S_R protein was cleaved by TEV enzyme and confirmed by western-blot analysis using anti-His antibody.

b, The cleavage patterns of CTSL on S_{2P} protein and S_R proteins. All samples were subjected to SDS-PAGE, and bands were detected by Coomassie blue staining.

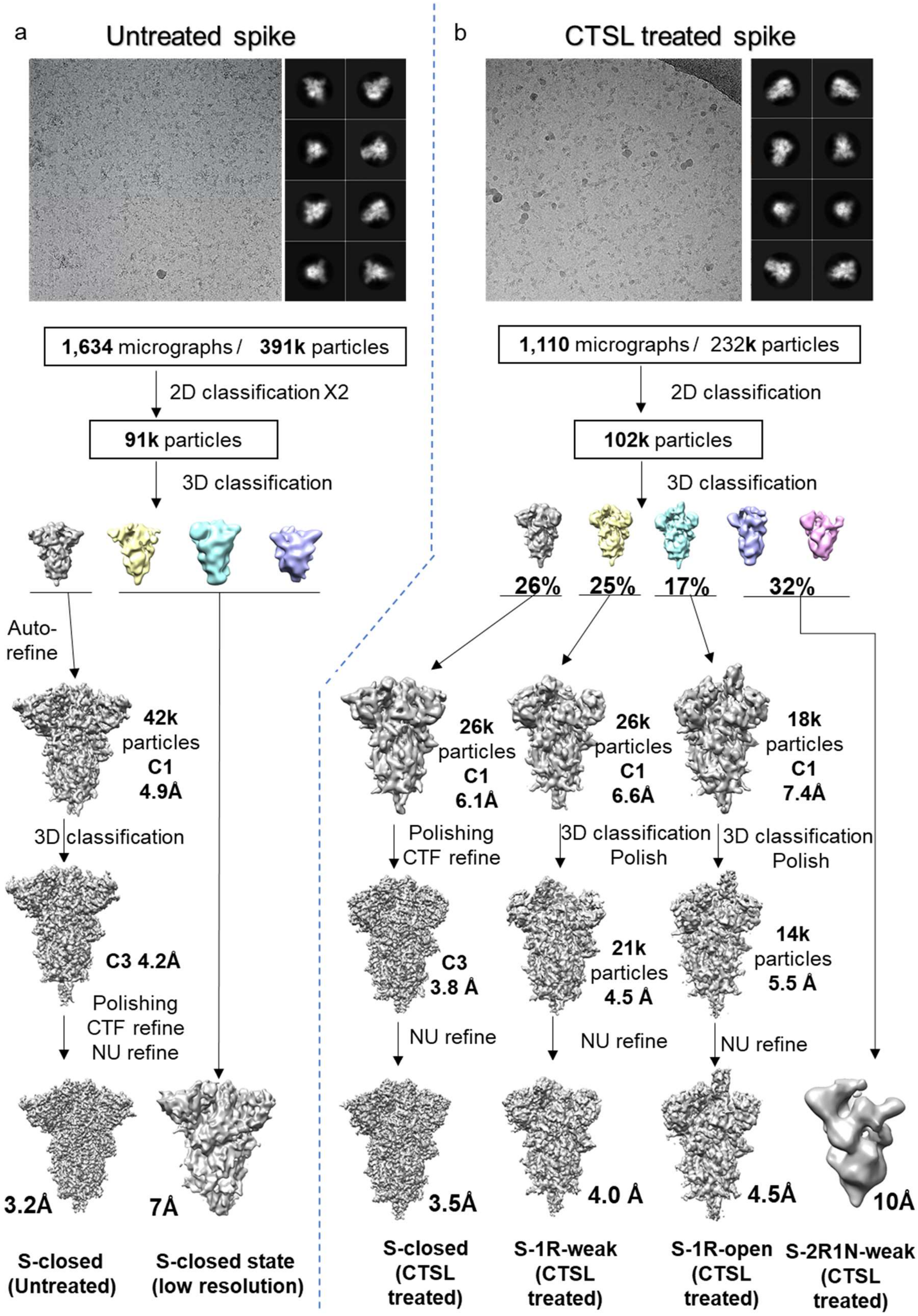

Supplementary Fig. S4. Single particle analysis workflow of untreated (a) or CTSL-treated (b) SARS-CoV-2 S.

Representative cryo-EM micrograph of the SARS-CoV-2 S protein and representative 2D class averages obtained from reference-free 2D classification are shown.

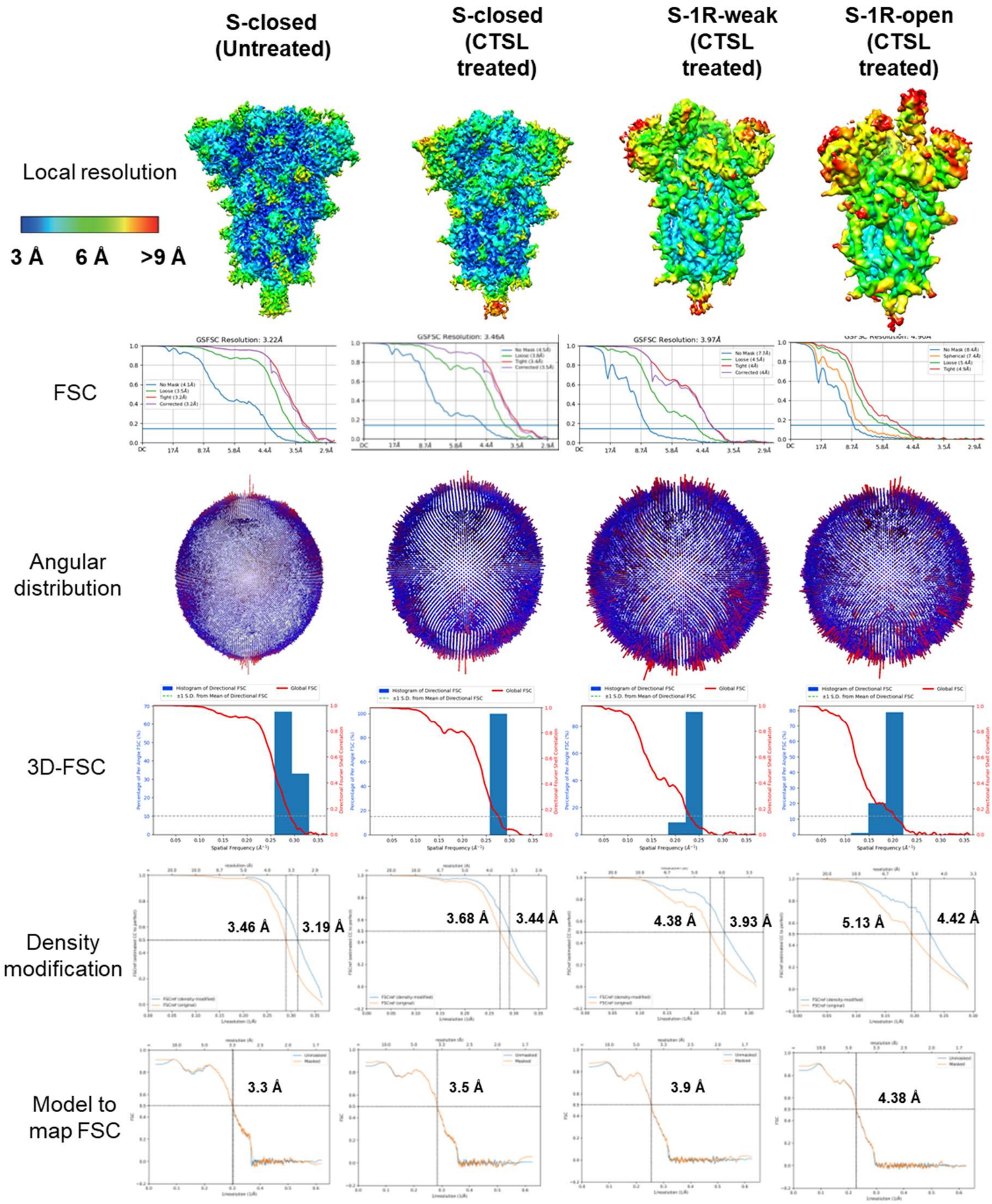

Supplementary Fig. S5. Cryo-EM map quality analysis of untreated or CTSL-treated SARS-CoV-2 S.

The local resolution analysis, gold-standard Fourier shell correlation (GSFSC), angular distribution, directional FSC (3DFSC), density modification and model-to-map FSC curves are shown.

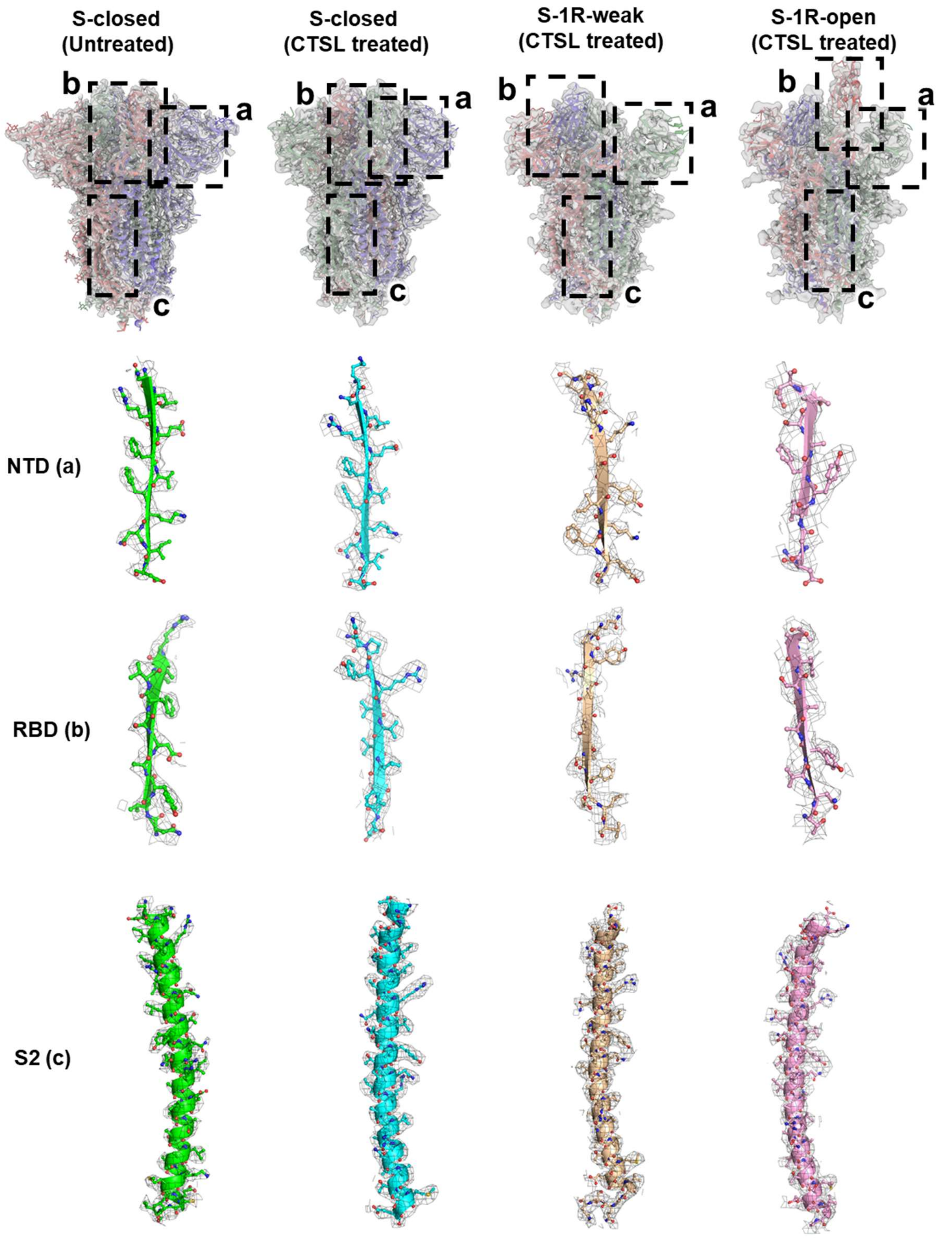

Supplementary Fig. S6. Model building quality of untreated or CTSL-treated SARS-CoV-2 S.

The structures of SARS-CoV-2 S in different states are fitted in the corresponding cryo-EM maps. The maps are shown in gray with transparency for the overall structure, or shown as black grid for the local regions.

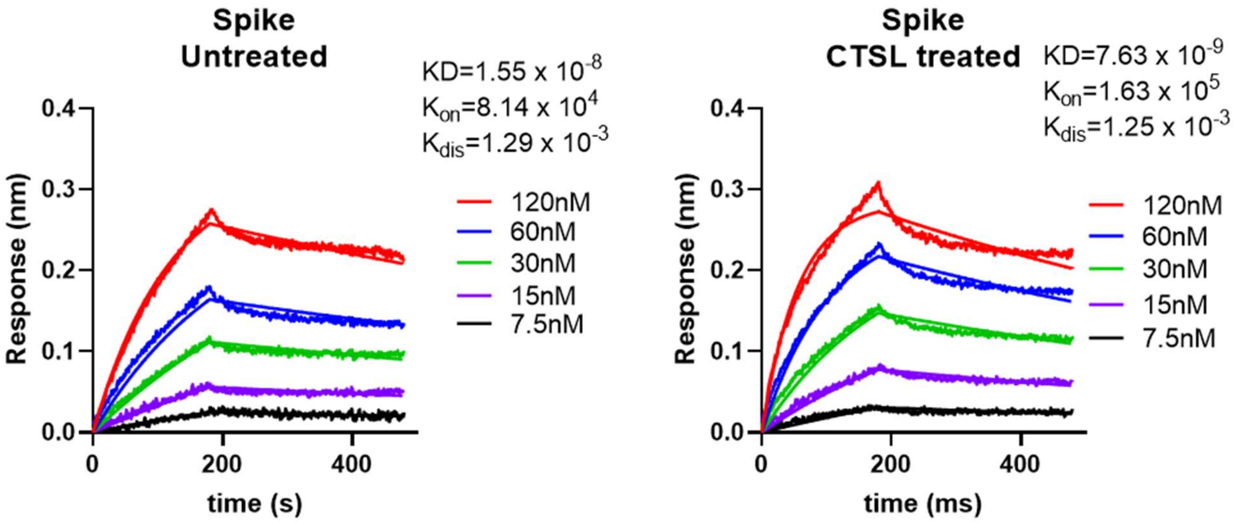

Supplementary Fig. S7. The binding affinities of the hACE2 to SARS-CoV-2 S proteins before and after CTSL treatment measured by BLI. The human IgG Fc-conjugated hACE2 protein was loaded for 300 s onto Anti-Human IgG Fc Capture (AHC) biosensors and tested for binding with gradient concentrations of purified S_R proteins under pH = 5.5. These experiments were repeated three times with similar results.

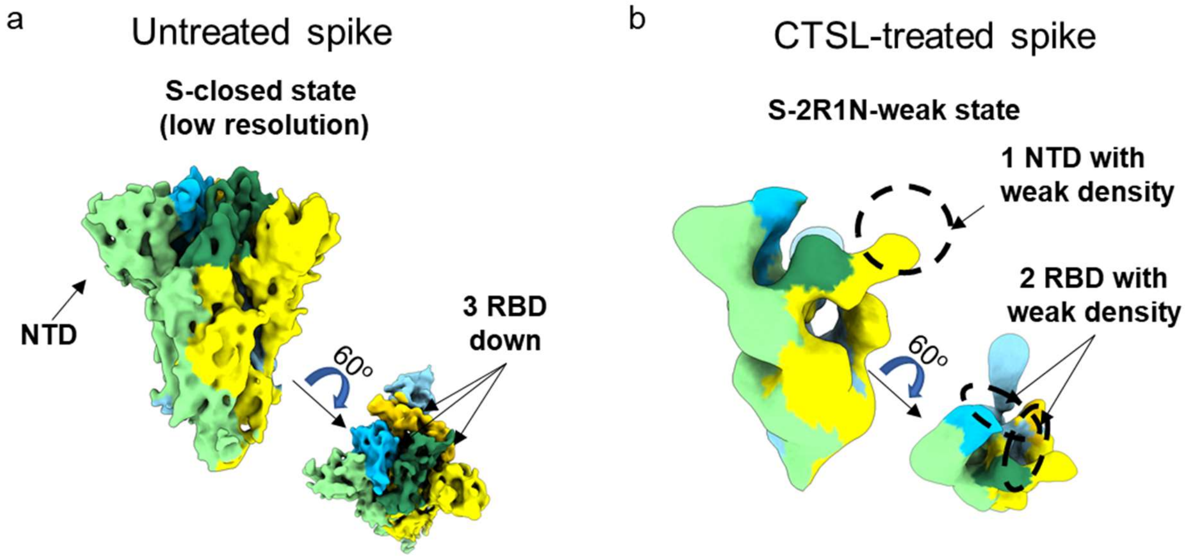

Supplementary Fig. S8. Structures of two low-resolution populations in untreated or CTSL-treated SARS-CoV-2 S.

Cryo-EM structures of SARS-CoV-2 S in S-closed state with low resolution of untreated group (**a**) and in S-2R1N-weak state of CTSL-treated group (**b**). Both side and top views are shown. The three protomers of S protein are shown in light green, yellow and sky blue. The three RBD domains are highlighted in sea green, gold and deep sky blue, respectively. RBD and NTD regions are indicated, and the region with weak density is shown in dashed circle.

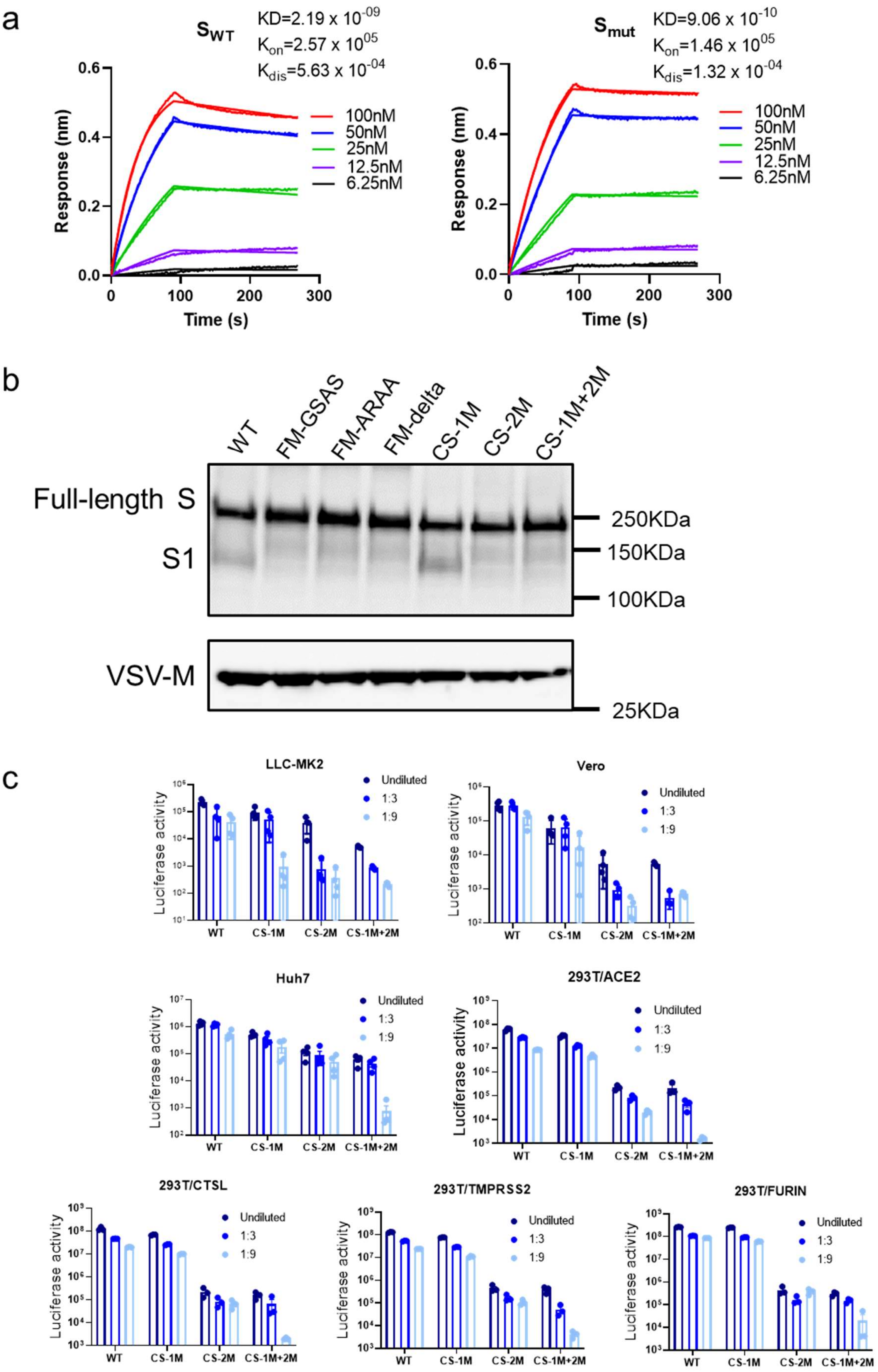
a
S_WT
KD=2.19 x 10^-09
K_on=2.57 x 10^05
K_dis=5.63 x 10^-04
S_mut
KD=9.06 x 10^-10
K_on=1.46 x 10^05
K_dis=1.32 x 10^-04
Response (nm)
Time (s)
100nM
50nM
25nM
12.5nM
6.25nM
b
WT
FM-GSAS
FM-ARAA
FM-delta
CS-1M
CS-2M
CS-1M+2M
Full-length S
S1
250KDa
150KDa
100KDa
VSV-M
25KDa
c
LLC-MK2
Vero
Huh7
293T/ACE2
293T/CTSL
293T/TMPRSS2
293T/FURIN
Luciferase activity
Undiluted
1:3
1:9
WT
CS-1M
CS-2M
CS-1M+2M

Supplementary Fig. S9. Validation and infection of mutant SARS-CoV-2 PsV.

a, Binding affinities of the hACE2 to the soluble WT S_{2P} and CS mutant (CS-1M+2M) S_{2P} proteins measured by BLI in Octet RED96 System. The human IgG Fc-conjugated hACE2 protein was loaded for 300 s onto Anti-Human IgG Fc Capture (AHC) biosensors and tested for binding with gradient concentrations of the indicated soluble S proteins under pH = 5.5.

b, Western blot analysis of different PsVs with anti-S1 and anti-VSV-M antibodies. VSV-M was used as the loading control.

c, Infectivity of PsVs at the indicated fold dilutions in in LLC-MK2, Vero, Huh7, 293T/ACE2 cells and in 293T/ACE2 cells with CTSL (293T/CTSL), TMPRSS2 (293T/TMPRSS2) and FURIN (293T/FURIN) genes overexpression. (n = 4).

The data are presented as the mean ± s.e.m. values.

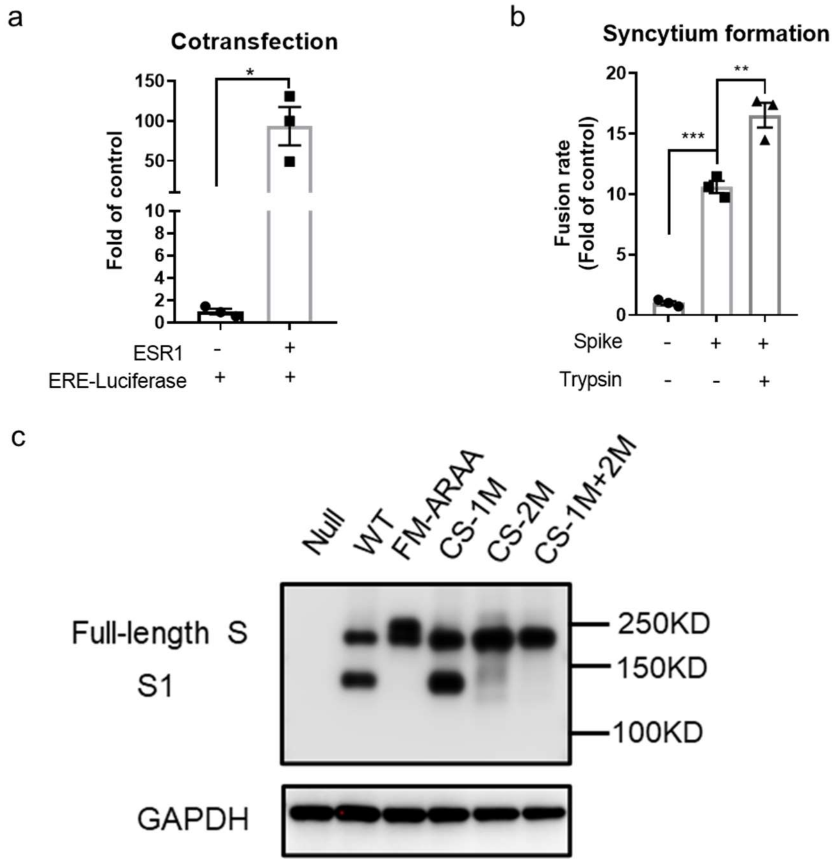

Supplementary Fig. S10. Quantification of syncytium formation.

a, ESR1 activated ERE-luciferase expression. Huh7 cells were cotransfected with the ERE-luciferase expression plasmid plus the scramble vector (control) or ESR1 plasmid. Luciferase activity was measured 24 h post transfection. The data were normalized as folds of control (*n* = 3). Statistical significance was assessed by unpaired two-tailed Student's *t* test.

b, SARS-CoV-2 S protein induced syncytium formation, which was quantified as the luciferase activity. In the trypsin-treated group, effector cells were added to target cells for 30-60 min, and the mixed cells were then treated with 2 μg/mL trypsin for 20 min. Then, the reaction was stopped by adding 500 μL of medium. Luciferase activity was measured after 24 h ($n = 3$). Statistical significance was assessed by one-way ANOVA with Tukey's post hoc test.

c, Huh7 cells transfected with different S plasmid or scramble control plasmid (null) as indicated. Western blot analysis of the cell lysates with anti-S1 and anti-GAPDH antibodies. GAPDH was used as the loading control.

The data are presented as the mean ± s.e.m. values. * $P < 0.05$, ** $P < 0.01$, ** $P < 0.001$.

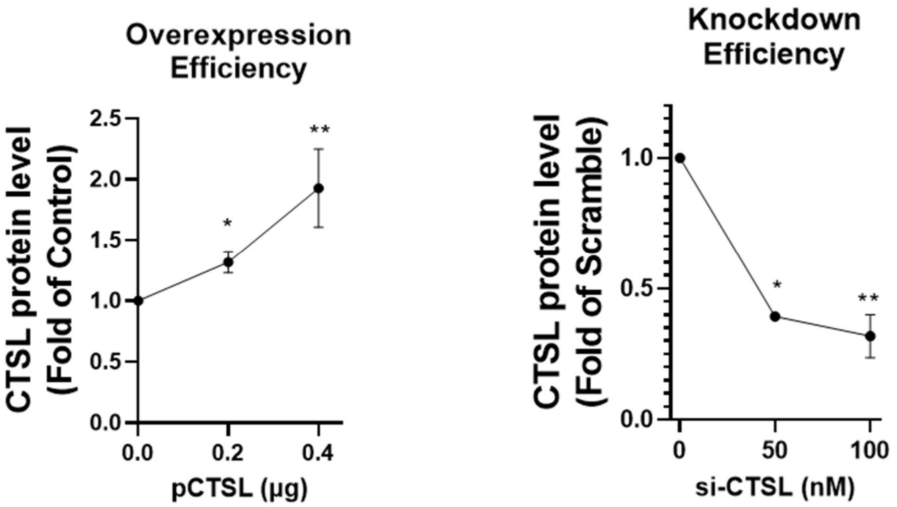

Supplementary Fig. S11. Validation of the overexpression and knockdown efficiency.

a, Dose-dependent knockdown of CTSL by siRNAs at the protein level by ELISA assay. $n = 4$. Statistical significance was assessed by the Kruskal-Wallis test with Dunn's post hoc test.

b, Dose-dependent overexpression of CTSL with a plasmid encoding the CTSL gene at the protein level by ELISA assay. $n = 5$. Statistical significance was assessed by the Kruskal-Wallis test with Dunn's post hoc test.

The data are expressed as the mean ± s.e.m. values. $*P<0.05$, $**P < 0.01$.

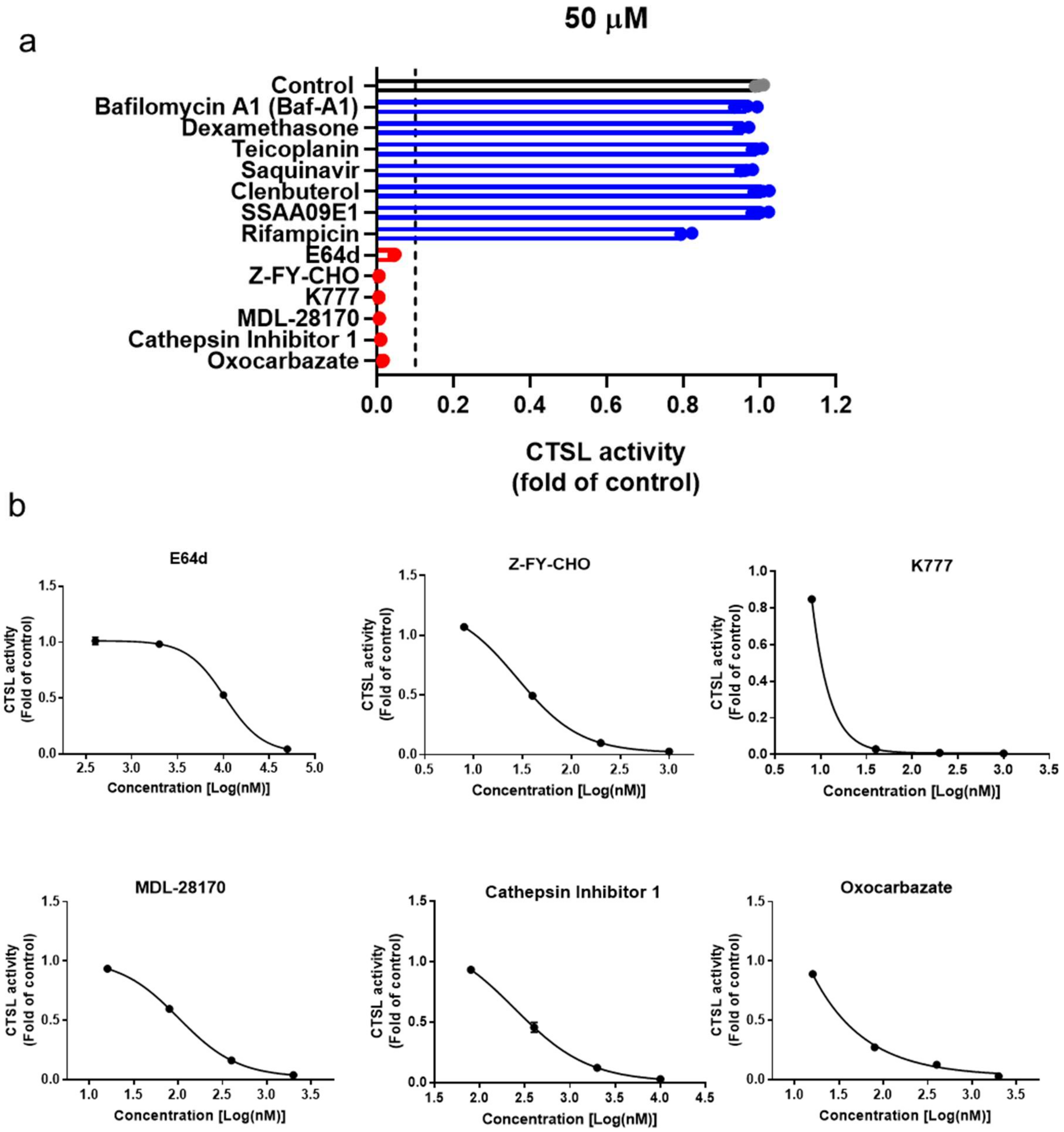

Supplementary Fig.12. Selection and validation of CTSL inhibitors.

a, The CTSL substrate Ac-FR-AFC (Abcam) was incubated with a total of 13 different drugs at 50 μM or with the same volume of vehicle. CTSL cleaves the substrate to release free AFC, which can be measured in a spectrophotometer at λ = 505 nm. After 1-2 h of incubation at 37 °C, CTSL activity was measured. The data were normalized to the vehicle-treated (control) group and presented as folds of control (*n* = 3).

b, CTSL activity was measured in the presence or absence of the 6 drugs at the indicated concentrations. The data were normalized to the vehicle-treated (control) group and are presented as folds of control (*n* = 3). The data are presented as the mean ± s.e.m. values.

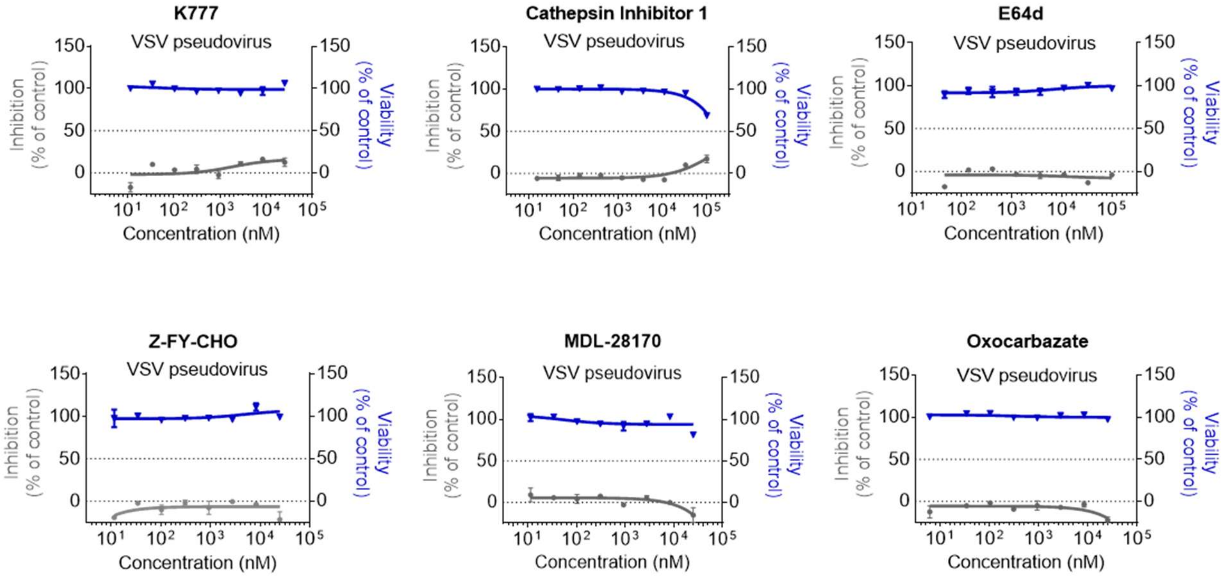

Supplementary Fig. S13. CTSL inhibitors do not affect the control of VSV PsV infection. Vero E6 cells were pretreated with increasing concentrations of each compound for 16 h and were then infected with VSV PsV. At 24 h post infection, infectivity was measured by a luciferase assay. The data were normalized to the average value in vehicle-treated cells and are shown as inhibition rates. Cell viability was evaluated with a CCK-8 kit (TransGen Biotech) (n = 3).

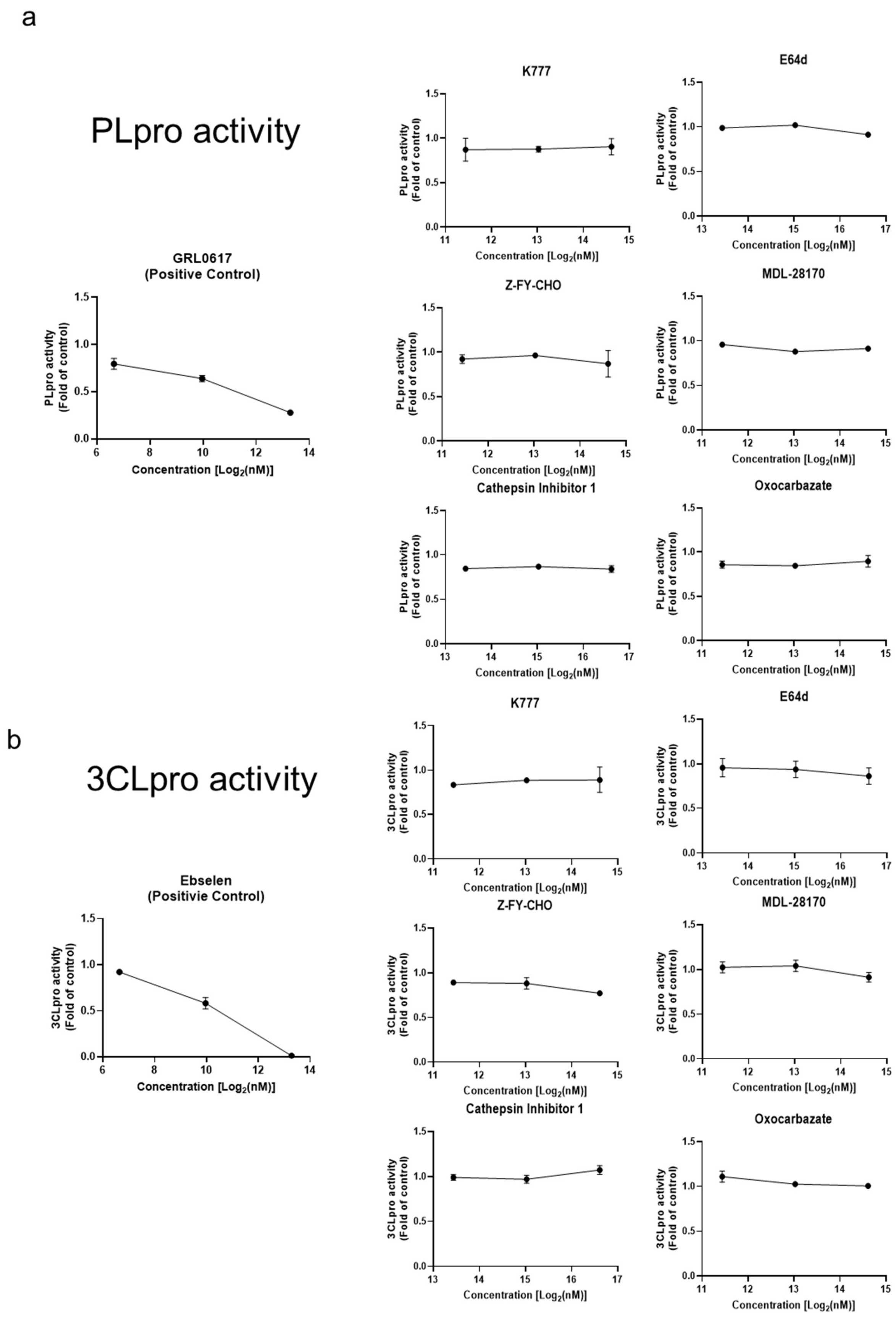

Supplementary Fig. S14. The effects of CTSL inhibitors to viral PLpro and 3CL pro activity.

a, The PLpro substrate Z-RLRGG-AMC (TGpeptide) was incubated with PLpro and the indicated drugs or with the same volume of vehicle. GRL0617, a PLpro inhibitor, served as a positive control. The highest concentrations of six CTSL inhibitors were same as the in vitro Vero E6 infection study. After 1-2 h of incubation, PLpro activity was measured. The data were normalized to the vehicle-treated (control) group and presented as folds of control (*n* = 3).

b, The 3CLpro substrate MCA-AVLQSGFR-Lys(Dnp)-Lys-NH_2 (Beyotime) was incubated with 3CLpro and the indicated drugs or with the same volume of vehicle. Ebselen, a 3CLpro inhibitor, served as a positive control. The highest concentrations of six CTSL inhibitors were same as the in vitro Vero E6 infection study. After 1-2 h of incubation, 3CLpro activity was measured. The data were normalized to the vehicle-treated (control) group and presented as folds of control (*n* = 3). The data are presented as the mean ± s.e.m. values.

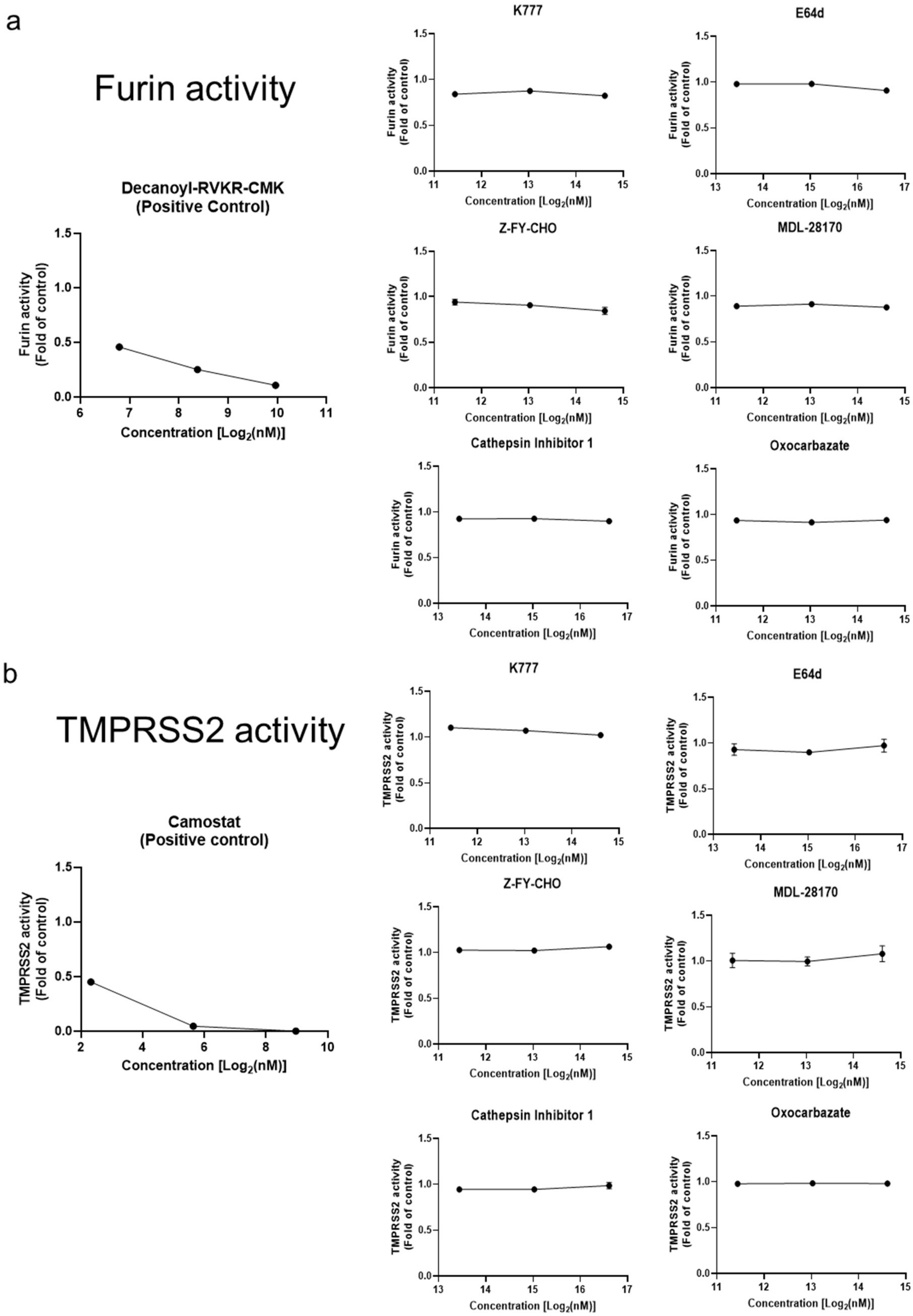

Supplementary Fig. S15. The effects of CTSL inhibitors to furin and TMPRSS2 activity.

a, The furin activity was measured using a commercial kit (AnaSpec) following the manufacturer. Decanoyl-RVKR-CMK, a furin inhibitor, served as a positive control. The highest concentrations of six CTSL inhibitors were same as the in vitro Vero E6 infection study. After 1-2 h of incubation, furin activity was measured. The data were normalized to the vehicle-treated (control) group and presented as folds of control (n = 3).

b, The TMPRSS2 substrate BOC-QAR-AMC (Absin) was incubated with TMPRSS2 and the indicated drugs or with the same volume of vehicle. Camostat, a TMPRSS2 inhibitor, served as a positive control. The highest concentrations of six CTSL inhibitors were same as the in vitro Vero E6 infection study. After 1-2 h of incubation, TMPRSS2 activity was measured. The data were normalized to the vehicle-treated (control) group and presented as folds of control (n = 3). The data are presented as the mean ± s.e.m. values.

Supplementary Table S1. RMSD values (angstrom) between different structural models of SARS-CoV-2 S proteins.

	**S-closed (CTSL treated)**	**S-1R-weak (CTSL treated)**	**S-1R-open (CTSL treated)**
**S-closed (untreated)**	0.677	1.277	1.122
**S-closed (CTSL treated)**	/	0.899	1.083
**S-1R-weak (CTSL treated)**	/	/	0.935

Supplementary Table S2. Statistics of cryo-EM data collection, image processing and model building of untreated and CTSL treated SARS-CoV-2 S.

**Sample**	**Untreated S**		**CTSL treated S**	
**Data Collection**				
Microscope	Titan Krios G2			
Voltage (kV)	300			
Detector	Gatan K2			
Energy filter	Gatan GIF Quantum, 20 eV			
Mode	Super resolution			
Pixel size (Å)	0.68			
Exposure (e/Å²)	60			
Number of images	1634		1110	
Defocus range (μm)	-1 ~ -3			
Software	SerialEM			
**Reconstruction**				
Software	RELION-3.0 & cryoSPARC-3.0			
Data set	**S-closed (untreated)**	**S-closed (CTSL treated)**	**S-1R-weak (CTSL treated)**	**S-1R-open (CTSL treated)**
Final particle No.	42115	26275	21045	13523
Symmetry	C3	C3	C1	C1
Final resolution (Å)	3.2	3.5	4.0	4.5
Map pixel size (Å)	1.36	1.35	1.35	1.35
Sharpening B-factor (Å²)	94.0	82.5	61.5	67.8
**Model building**				
Software	Coot & Phenix			
Map CC (mask)	0.78	0.77	0.74	0.74
Rmsd (bonds) (Å)	0.002	0.002	0.005	0.003
Rmsd (angle) (°)	0.670	0.694	0.666	0.610
Protein Residues	3309	3165	2880	2878
Ramachandran outliers (%)	0.00	0.48	0.32	0.07
Ramachandran allowed (%)	3.2	3.95	5.50	5.17
Ramachandran favored (%)	96.8	95.57	94.18	94.76
Rotamer outliers (%)	0	0	0	0

Supplementary Table S3. siRNA sequence mixture against human CTSL

Dulex Name	Sense Seq (5'-3')	Anti Seq (5'-3')
**CTSL-1 (human)**	AGGCGAUGCACAACAGAUUAUTT	AUAAUCUGUUGUGCAUCGCCUTT
**CTSL-2 (human)**	CCAAAGACCGGAGAAACCAUUTT	AAUGGUUUCUCCGGUCUUUGGTT
**CTSL-3 (human)**	GUGGGAGAAGAACAUGAAGAUTT	AUCUUCAUGUUCUUCUCCCACTT
**CTSL-4 (human)**	AGGAGAAGGCCCUGAUGAATT	UUCAUCAGGGCCUUCUCCUTT
**CTSL-5 (human)**	GGAUUAUGCUUUCCAGUAUTT	AUACUGGAAAGCAUAAUCCTT
**CTSL-6 (human)**	GGGCAUGGGUGGCUACGUATT	UACGUAGCCACCCAUGCCCTT

Supplementary Video S1-4.

Supplementary Video S1. Overall map of SARS-CoV-2 S protein in untreated S-closed state with model fitted. Views are sliced to show the model and map quality.

Supplementary Video S2. Overall map of SARS-CoV-2 S protein in CTSL-treated S-closed state with model fitted. Views are sliced to show the model and map quality.

Supplementary Video S3. Overall map of SARS-CoV-2 S protein in CTSL-treated S-1R-weak state with model fitted. Views are sliced to show the model and map quality.

Supplementary Video S4. Overall map of SARS-CoV-2 S protein in CTSL-treated S-1R-open state with model fitted. Views are sliced to show the model and map quality.
